# Transcript profiling for early stages during embryo development in Scots pine

**DOI:** 10.1186/s12870-016-0939-5

**Published:** 2016-11-18

**Authors:** Irene Merino, Malin Abrahamsson, Lieven Sterck, Blanca Craven-Bartle, Francisco Canovas, Sara von Arnold

**Affiliations:** 1 Department of Plant Biology, Uppsala BioCenter, Swedish University of Agricultural Sciences, Box 7080, 750 07 Uppsala, Sweden; 2 Department of Plant Systems Biology, VIB, B-9052 Ghent, Belgium; 3 Department of Plant Biotechnology and Bioinformatics, Ghent University, B-9052 Ghent, Belgium; 4 Bioinformatics Institute Ghent, Ghent University, Ghent, B-9000 Belgium; 5 Department of Molecular Biology and Biochemistry, School of Sciences, Campus de Teatinos, Universidad de Malaga, 29071 Malaga, Spain

**Keywords:** Embryo development, *Pinus sylvestris* L, Polyembryony, RNA-seq, Transcriptomic analysis, Zygotic embryogenesis

## Abstract

**Background:**

Characterization of the expression and function of genes regulating embryo development in conifers is interesting from an evolutionary point of view. However, our knowledge about the regulation of embryo development in conifers is limited. During early embryo development in *Pinus* species the proembyo goes through a cleavage process, named cleavage polyembryony, giving rise to four embryos. One of these embryos develops to a dominant embryo, which will develop further into a mature, cotyledonary embryo, while the other embryos, the subordinate embryos, are degraded. The main goal of this study has been to identify processes that might be important for regulating the cleavage process and for the development of a dominant embryo.

**Results:**

RNA samples from embryos and megagametophytes at four early developmental stages during seed development in *Pinus sylvestris* were subjected to high-throughput sequencing. A total of 6.6 million raw reads was generated, resulting in 121,938 transcripts, out of which 36.106 contained ORFs. 18,638 transcripts were differentially expressed (DETs) in embryos and megagametophytes. GO enrichment analysis of transcripts up-regulated in embryos showed enrichment for different cellular processes, while those up-regulated in megagametophytes were enriched for accumulation of storage material and responses to stress. The highest number of DETs was detected during the initiation of the cleavage process. Transcripts related to embryogenic competence, cell wall modifications, cell division pattern, axis specification and response to hormones and stress were highly abundant and differentially expressed during early embryo development. The abundance of representative DETs was confirmed by qRT-PCR analyses.

**Conclusion:**

Based on the processes identified in the GO enrichment analyses and the expression of the selected transcripts we suggest that (i) processes related to embryogenic competence and cell wall loosening are involved in activating the cleavage process; (ii) apical-basal polarization is strictly regulated in dominant embryos but not in the subordinate embryos; (iii) the transition from the morphogenic phase to the maturation phase is not completed in subordinate embryos. This is the first genome-wide transcript expression profiling of the earliest stages during embryo development in a *Pinus* species. Our results can serve as a framework for future studies to reveal the functions of identified genes.

**Electronic supplementary material:**

The online version of this article (doi:10.1186/s12870-016-0939-5) contains supplementary material, which is available to authorized users.

## Background

Post-embryonic development in plants depends on the establishment of stem cell niches in shoot and root meristems that take place during embryogenesis. Pattern formation in the embryo is under the control of co-ordinated spatially and temporally regulated gene expression, cell division, and hormone function. Most of our knowledge about the regulation of pattern formation during embryo development is based on studies of embryo-defective mutants in the angiosperm model plant Arabidopsis (*Arabidopsis thaliana*) [1]. By contrast, our knowledge about the regulation of embryo development in gymnosperms is limited. Molecular data suggest that extant seed plants (gymnosperms and angiosperms) shared a final common ancestor about 300 million years ago [2]. Therefore, characterization of the expression and functions of genes regulating embryo development in gymnosperms is interesting from an evolutionary point of view. Another reason to study gymnosperms, and especially conifers, is that they are of great commercial importance.

Embryo development in *Pinus* can be divided into three phases [3]: (1) proembryogeny – all stages before elongation of the suspensor, (2) early embryogeny – all stages during and after elongation of the suspensor and before establishment of the root meristem, (3) late embryogeny – establishment of the root and shoot meristem and further development of the embryo. Proembryogeny starts with a free nuclear stage. The zygote undergoes several rounds of nuclear duplication that are not followed by cytokinesis. After cell wall formation, four tiers are formed of which the lowest tier will form the embryonal mass and the second lowest tier will elongate to form the embryonal suspensor. In most *Pinus* species the four apical cells and the suspensor network in the proembryo separate into four filamentous embryos [4, 5]. This process is termed cleavage polyembryony [6]. The four embryos, which arise from the separated tiers, begin their development by apical cell growth [7]. The basal cells of the embryonal mass divide anticlinally and elongate, contributing to the suspensor, which consists of several files of terminally differentiated non-dividing cells. Early embryogeny begins with the elongation of the suspensor. The enlarging suspensor pushes the embryo out of its archegonial jacket into the rich nutritive reserves of the megagametophyte. In an ovule where polyembryony is present, the competition between genetically identical embryos offers no selective advantage; instead it has been suggested that the embryo with the best physiological constitution situated in the most suitable environment becomes the dominant embryo, which usually develops to maturity [8]. The rest of the embryos, the subordinate embryos, are degraded by programmed cell death (PCD) [9]. During late embryogeny the root and shoot apical meristems are delineated and the plant axis established. The maturing embryo is characterized by the initiation of cotyledons.

Various approaches have been taken for elucidating the regulation of embryo development in plants. The most comprehensive study of transcript profiling in a conifer was conducted in loblolly pine (*Pinus taeda*) where approximately 68,700 ESTs were regenerated from zygotic and somatic embryos [10]. Based on 295 genes, essential for embryogenesis in Arabidopsis, 85% had very strong sequence similarity to an EST in the loblolly pine database [11]. Stress-related processes and auxin-mediated-processes were, by using microarray analysis, identified to be associated with early somatic embryo development in Norway spruce [12]. Microarray analysis has also been performed for studying global gene expression during development of dominant zygotic embryos of maritime pine (*Pinus pinaster*) [13]. The results revealed that epigenetic regulation and transcriptional control related to auxin transport and response are critical during early to mid-stages of pine embryogenesis, and that important events during embryogenesis seem to be coordinated by putative orthologs of major developmental regulators in angiosperms. Recent advances in high-throughput sequencing technologies enable global transcriptome profiling without prior sequence knowledge [14]. By analysing the transcription network between embryo and endosperm during early seed development in maize (*Zea mays*) it was shown that many metabolic activities are specific for the embryo or the endosperm, and that transcription factors and imprinting genes are specifically expressed in the embryo or the endosperm [15]. Comparative transcriptome analysis of somatic and zygotic embryos in cotton (*Gossypium hirsutum*) uncovered that the process of somatic embryogenesis is characterized by induction of several stress-related genes [16]. Whole transcriptome profiling during initiation of embryogenic tissue in maize showed an increased expression of stress factors and the importance of a coordinated expression of somatic embryogenesis-related genes [17], as well as the involvement of a complex auxin-signalling pathway [18]. Several metabolic events were detected by transcriptome analysis in proliferating embryogenic cultures of Japanese larch (*Larix leptolepis*) [19].

To improve the understanding of genomic factors involved in early embryo development in Scots pine we performed a genome-wide high-throughput transcriptome sequencing for early stages during zygotic embryogenesis. The expression of twenty-three differentially expressed genes was confirmed by qRT-PCR analyses. Based on these analyses, and on the assumption that the Scots pine genes are homologous to their Arabidopsis counterparts, we have identified transcripts and putative processes that take place during early embryo development including initiation of cleavage polyembryogeny and development of dominant embryos. To our knowledge, this represents the first genome-wide transcript expression profiling of the earliest stages during embryo development in a *Pinus* species.

## Methods

### Plant material

**Fig. 1:**
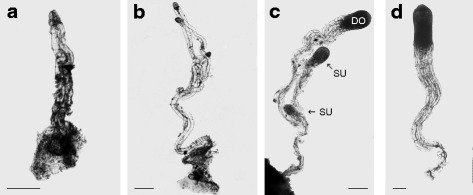
Early stages during development of zygotic embryos in Scots pine that have been included in this study. **a** A single zygote-derived early embryo (stage 1; E1). **b** The single embryo at stage E1 has cleaved to form multiple embryos of equal size (stage 2; E2). **c** One embryo has become dominant (stage 3; E3DO) and subordinate embryos (stage 3; E3SU) successively stop developing. **d** A well-developed dominant embryo before cotyledon differentiation (stage 4; E4). Bars 0.5 mm

Immature cones were collected for sequencing during summer 2012, from an open-pollinated seed orchard clone (W4009) of Scots pine (*Pinus sylvestris* L.) in Hade, central Sweden (60.3° latitude, 17.0° longitude). The Swedish Forestry Research Institute, that is running the seed orchard, had given us permission to collect cones. The same clone has been used for sequencing the Scots pine genome (WP1 in the European Community’s Seventh Framework Programme, ProCoGen project). In order to collect the desired developmental stages of zygotic embryos and megagametophytes, cones were harvested periodically between the 11^th^ and 20^th^ of July. The zygotic embryos were excised from the megagametophytes under a stereomicroscope. Both the embryos (E) and the megagametophytes (M) were sorted and collected, in Eppendorf tubes placed on ice, according to developmental stage (Fig. 1): Stage 1, the ovules contained a single embryo at the stage before cleavage (E1, M1); Stage 2, the ovules contained an embryo at the stage of cleavage (E2, M2); Stage 3, the ovules contained a dominant embryo, DO, and subordinate embryos, SU (the dominant and the subordinate embryos were sampled separately, E3DO, E3SU, M3); Stage 4, the ovule contained a dominant embryo just before cotyledon differentiation (E4, M4). After a maximum of 10 min on ice, the Eppendorf tubes with collected embryos or megagametophytes were frozen in liquid nitrogen. Each sample included from 27 to 50 embryos, depending on the developmental stage. Equivalent materials were collected for qRT-PCR analyses during summer 2014. To avoid specificity of the embryogenesis process in a particular region, the new samples were collected from a tree of Scots pine growing at the SLU estate located in Ultuna, central Sweden (59.8° latitude, 17.7° longitude), from which an unlimited number of cones could be collected. The harvesting of cones was performed periodically between the 17^th^ of June and 8^th^ of July.

### RNA extraction and cDNA synthesis

Total RNA from zygotic embryos was extracted using the RNAqueous-Micro RNA Isolation Kit (Ambion), followed by a DNase I treatment to remove any residual genomic DNA, according to manufacturer’s instructions. RNA from megagametophytes was isolated using the Spectrum Plant Total RNA kit (Sigma-Aldrich), including the On-Column DNAse I Digestion step for removing traces of genomic DNA.

The concentration of the isolated RNA from samples collected for RNA sequencing (one biological replicate) was determined fluorometrically using a Qubit fluorometer (Invitrogen), and the integrity was verified by an Agilent BioAnalyzer using the RNA 6000 Nano chip (Agilent Tecnologies). The RNA samples with an RNA integrity number (RIN) higher than 7 were used for cDNA synthesis and amplification with the Mint-2 cDNA synthesis kit (Evrogen). Briefly, first-strand synthesis was initiated from 1 μg of total RNA by a Mint RT using a modified poly-dT primer. Second strand synthesis was carried out by the Encyclo DNA polymerase (Evrogen), followed by PCR amplification. The number of cycles (18 or 21) for double-strand cDNA (dsDNA) amplification was estimated for each sample. Amplified cDNA was then purified with the NucleoSpin Gel and PCR Clean-up kit (Macheray-Nagel). Finally, a reamplification step was performed with specific primers for 454 pyrosequencing. In total, nine different dsDNA enriched libraries were constructed, five from zygotic embryos at stage E1, E2, E3DO, E3SU and E4, and four from megagametophytes at stage M1, M2, M3 and M4.

RNA isolated from embryos collected for qRT-PCR (four biological replicates) was quantified using a NanoDrop-1000 spectrophotometer (Nanodrop Technologies). cDNA synthesis from 100 ng of total RNA was performed using the QuantiTect Whole Transcriptome (Qiagen) followed by 8 h amplification according to the manufacturer’s protocol for high-yield reactions.

### Transcriptome sequencing

Transcriptome sequencing was performed at the University of Malaga ultrasequencing facility using the GS-FLX + platform with a GS-FLX Titanium kit, Roche Applied Sciences (Indianapolis, IN, USA) following the protocol described by Canas et al. [20].

### Transcript reconstruction from RNA-seq

Before assembly, the 6.6 million raw 454 reads were quality checked, and short reads (<75 bp) and adapter sequences were removed from the dataset using seqclean (Additional file 1: Table S1). After cleaning the reads were *de novo* assembled using the Newbler software (v2.8.1) which resulted in 76,425 isogroups containing 117,551 isotigs (Additional file 1: Table S2). In order to get an even more comprehensive transcriptome dataset, we then also incorporated publically available datasets with the previously obtained assembly. We integrated another 67,744 PUTs from PlantGDB (Resources for Comparative Plant Genomes) [21] and a set of 2161 ESTs from the NCBI ESTdb, which were used as a reference to map reads against (Additional file 1: Table S3). The various datasets were integrated using the CD-HIT software [22] in order to remove redundant transcripts and clustering into isogroups. For each isogroup only the longest isotig was retained for further analysis. This resulted in a final transcriptome set of 121,938 transcripts (Additional file 1: Table S3). The lengths of the assembled transcripts are shown in Additional file 1: Table S4.

Note: PUTs and Cl/118 transcripts that were not present in the seed transcriptome did not receive any reads, so their RPKM was 0 for all the stages and were then removed from the differential expression analysis.

For expression quantification of each sample, all cleaned reads were mapped back to the integrated transcript set using BWA [23]. Afterwards the mapping results were processed with samtools [24] to obtain read counts, which were then processed with an in-house PERL script to result in RPKM values for each transcript.

Open Reading Frame (ORF) predictions on the total 121,938 transcripts were obtained by applying TransDecoder with default parameters except for the coding model, which was specific for *P. sylvestris*, built from manually curated full length transcripts. TransDecoder identified 36,106 ORFs in the dataset.

### Functional annotation and enrichment analysis

In order to functionally characterise the resulting ORFs, a blastP analysis (e-value cut-off 1e-5) was performed against the Arabidopsis TAIR10 database. All ORFs were also analysed for protein domains with interproscan (v5.13.52) [25] and possible GO-terms were determined based on the InterPro domains. To identify putative transcription factors (TFs) in our dataset all ORFs were screened against the Plant Transcription Factor Database, PlantTFDB v3.0 [26] using blastP (e-value cut-off 1e-5).

Gene annotation analyses and functional enrichment of differentially expressed transcripts in embryos and megagametophytes were performed with WeGO (Web Gene Ontology Annotation Plot) tool [27] and AgriGO analysis toolkit [28] respectively. For functional enrichment analyses the seed annotated transcriptome was used as background/reference genome. Hypergeometric test was used as statistical method with an adjusted FDR value (cut-off 0.05) and complete GO was selected as gene ontology type in the settings.

### Identification of differentially expressed transcripts

For identification of differentially expressed transcripts (DETs), pairwise comparisons were performed between: (i) embryos and megagametophytes at the same developmental stage (E1 vs M1, E2 vs M2, E3DO vs M3, E3SU vs M3 and E4 vs M4), (ii) embryos at consecutive developmental stages (E1 vs E2, E2 vs E3DO, E3DO vs E4, E3DO vs E3SU) and (iii) megagametophytes at consecutive developmental stages (M1 vs M2, M2 vs M3, M3 vs M4). The relative fold-change (FC) is presented as log2 of the RPKM ratio (sample A/sample B). Transcripts with a FC higher than 2 were considered as differentially expressed transcripts (DETs). When the RPKM value of one sample was 0 (no expression detected) the fold-change could not be estimated. In these cases 99 and -99 values were assigned as relative fold-changes. In addition, when the RPKM value was 0 in one of the samples and lower than 10 in the other, the transcript was excluded from differential expression analyses.

Venn diagrams have been drawn with the online web tool available at http://bioinformatics.psb.ugent.be/webtools/Venn/.

K-means cluster analysis was performed with a subset of DETs (FC higher than 2 and RPKM over 10) detected in any of the pairwise comparisons between different developmental stages in embryos and in megagametophytes. Initially, the relative expression value for each DET was calculated by normalizing all the RPKM values from different developmental stages to its maximum RPKM value. The optimal number of clusters was estimated separately for the embryo and megagametophyte data and normalized values were clustered using the kmeans function in R software.

### Quantitative RT-PCR

Quantitative RT-PCR was performed in a Bio-Rad CFX Connect™ Real-Time PCR Detection System cycler (Bio-Rad Laboratories). All samples were run in duplicate starting from 5 ng of cDNA from four biological replicates for each developmental stage. *ELONGATION FACTOR 1* (*EF1*) and *PHOSPHOGLUCOMUTASE* (*PHOS*) were used as reference genes [12]. Relative quantitative analyses were performed following the 2^-ΔΔCt^ Livak method. Only transcripts showing a similar expression profile in at least three out of four biological replicates have been included. The primer sequences for the transcripts tested are shown in Additional file 2: Table S5. Significant differences in transcript accumulation between different developmental stages were estimated by a *t-*test mean comparison analysis (*P* ≤ 0.05) using the JMP software (v11).

To validate the RNA-seq data, the same RNA that was used for sequencing was tested by qRT-PCR. New cDNA from embryos was synthesized and amplified using the QuantiTect Whole Transcriptome kit (Qiagen), as has been explained above. cDNA from megagametophytes was synthesized by using the PrimeScript™ RT reagent Kit (Takara), according to manufacturer’s instructions. The Pearson correlation coefficient between the expression profiles obtained by RNA-seq and qRT-PCR was calculated for each of the 23 candidate transcripts in embryos and for 7 selected DETs (involved in response to stress and stimulus) in megagametophytes (Additional file 2: Table S6). The correlation coefficient was estimated by using the Pearson statistical function in Microsoft Excel.

## Results and discussion

### Overview of the transcriptome in seeds

To identify transcripts and biological processes involved in early zygotic embryogenesis in Scots pine, RNA was isolated from embryos and megagametophytes representing four developmental stages (Fig. 1). Nine RNA-seq libraries were sequenced by using 454 Roche sequencing technology. A total of 6.6 million raw reads was generated, resulting in 121,938 transcripts varying in length from 150 to 18,101 bp and with a mean length of 1242 bp (Additional file 1: Tables S1, S2, S3 and S4).

In total, 36,106 transcripts containing ORFs were identified in the seed transcriptome, of which 28,190 transcripts (78%) had significant alignments to the *Arabidopsis thaliana* TAIR10 database and 7404 transcripts (20%) with the Plant Transcription Factor Database (Table 1). 26,743 transcripts (74%) had annotated GO terms into at least one of the three main categories: 22,362 transcripts (60%) displayed one or more ontologies related to Biological Process, 24,259 (67%) to Molecular Function and 19,301 (53%) to Cell Component.

**Table 1:** Summary of RNA-seq seed transcriptome data

	All samples	Embryo samples	Megagametophyte samples
Transcripts with RPKM > 0	81,120	74,149	59,524
Transcription factors	7404	7200	6605
Transcripts with ORFs	36,106	29,595	26,400
Transcripts with hits against TAIR database	28,190	24,043	22,556
Annotated transcripts (GO)	26,743	25,441	23,309

**Fig. 2:**
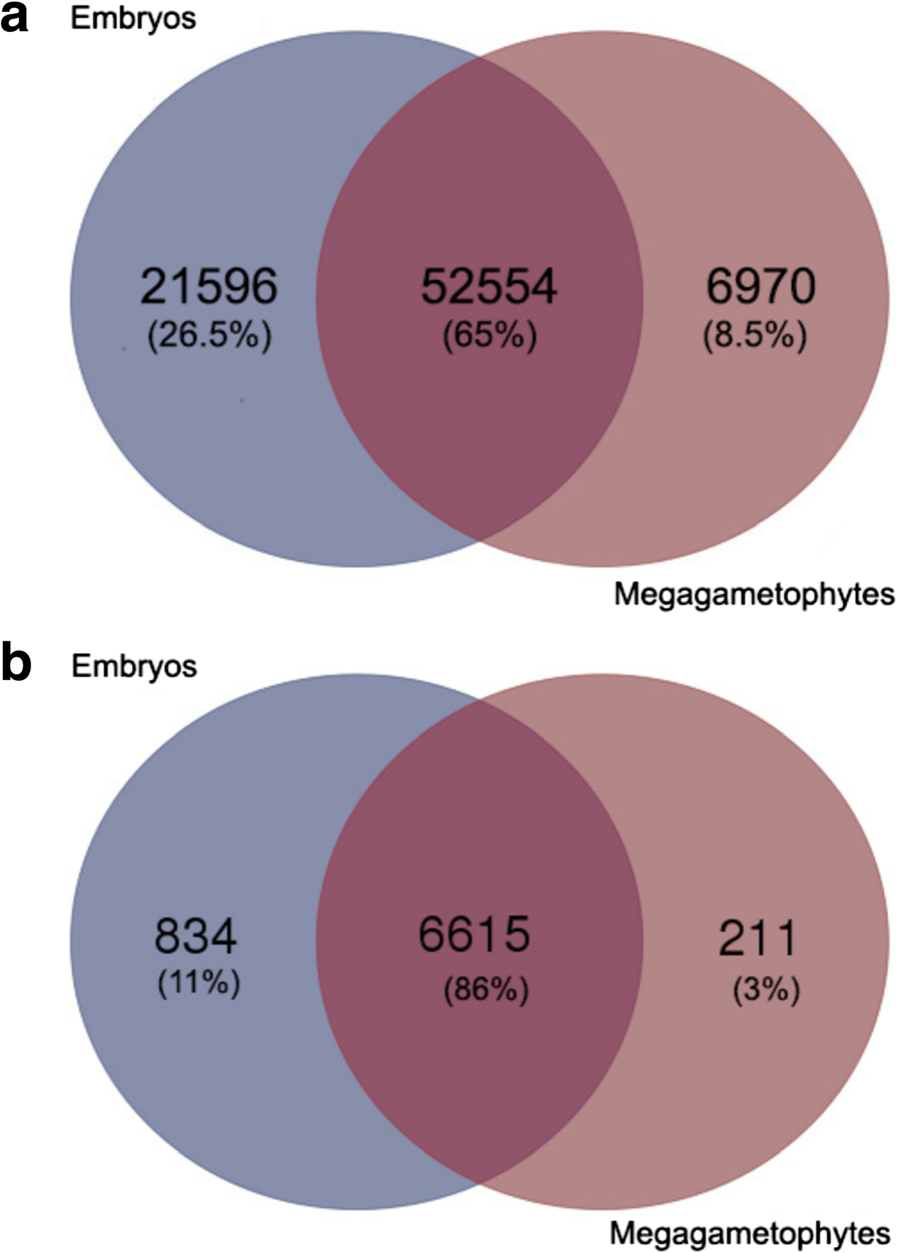
Venn diagram demonstrating the total number of transcripts and TFs detected in embryos and megagametophytes. Numbers in the intersection represent transcripts/TFs detected both in embryos and megagametophytes. **a** All detected transcripts (RPKM > 0) in the seed transcriptome. **b** Number of TFs (RPKM > 0) detected in the seed transcriptome

Transcript expression values were calculated as RPKM, resulting in 81,120 assembled transcripts with detectable expression signals (RPKM >0), in at least one of the developmental stage (Table 1). 74,150 and 59,526 transcripts were detected in embryos and megagametophytes, respectively. Most of the transcripts (65%) were detected in both tissues, however the number of unique transcripts was threefold higher in embryos than in megagametophytes (Fig. 2a). The number of identified transcription factors (TFs) was also higher in embryos (Fig. 2b).

The total number of transcripts detected at each developmental stage during seed development increased in embryos, but decreased in megagametophytes (Table 2). Around 15,000 transcripts were expressed at all developmental stages both in embryos and in megagametophytes. The number of unique transcripts detected at specific developmental stages was fairly constant in the embryos, but decreased in the megagametophytes during seed development from 10,907 transcripts at stage M1 to 3201 transcripts at stage M4 (Additional file 1: Figure S1A and B). Out of 7404 TFs identified during early embryo development, 3734 TFs (50%) were detected at all developmental stages, and about 140 TFs were only detected at one developmental stage (Additional file 1: Figure S1C). In megagametophytes, 3775 TFs (56%) were detected at all developmental stages, however, the number of TFs detected at only one developmental stage decreased during seed development (Additional file 1: Figure S1D).

**Table 2:** Number of transcripts and TFs detected in embryos and megagametophytes at different developmental stages (RPKM > 0)

Embryos	E1	E2	E3DO	E3RE	E4
Transcripts	39,423	43,309	46,976	46,492	47,089
TFs	4993	5725	5936	5641	5810
Megagametophytes	M1	M2	M3		M4
Transcripts	40,595	38,853	35,188		34,235
TFs	5468	5249	5073		4989

To test the reliability of the RNA-seq results, 30 transcripts (23 transcripts in embryos and 7 transcripts in megagametophytes) were selected for examination by qRT-PCR. The Pearson correlation coefficient between the expression profiles obtained by RNA-seq and qRT-PCR was calculated from each transcript separately (Additional file 2: Table S6). The correlation coefficient obtained was similar for most transcripts, except for a few transcripts at some time points.

### Changes in transcript accumulation during seed development

#### Differentially expressed transcripts in pairwise comparisons between embryos and megagametophytes during seed development

To identify differentially expressed transcripts (DETs) we performed pairwise comparisons between embryos and megagametophytes at the same developmental stage. In total 18,638 transcripts were up-regulated with a fold change higher than 2 (FC > 2) in at least one of the pairwise comparisons between embryos and megagametophytes (Additional file 3: Figure S2A, Additional file 4). 12,906 transcripts were up-regulated in embryos and 5732 in megagametophytes. The greatest difference in the number of up-regulated transcripts between embryos and megagametophytes was observed at developmental stage 2 (Additional file 3: Figure S2B).

**Fig. 3:**
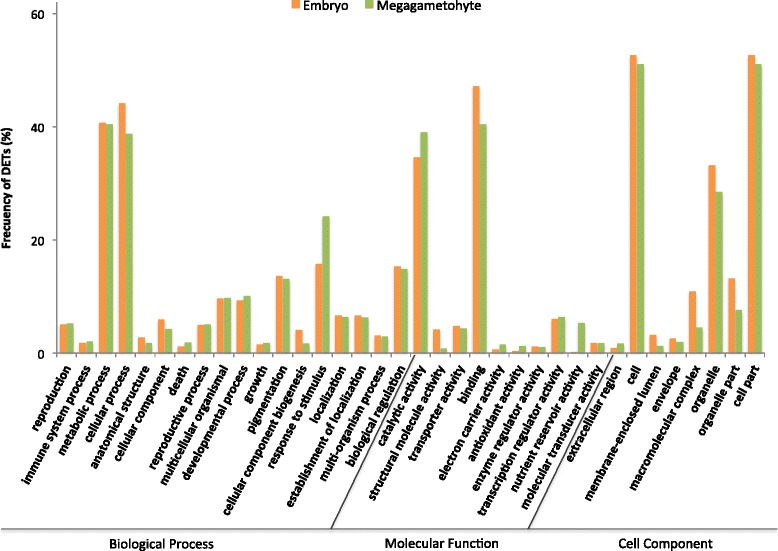
GO annotation analysis of differentially expressed transcripts (DETs) in embryos and megagametophytes. The analysis included the total number of DETs with a fold-change greater than 2 (FC > 2) in any of the pairwise comparisons between embryos and megagametophytes. Presented data show the percentage of transcripts related to the total number of transcripts used as input in each GO subcategory (level 2) in embryos (orange) and in megagametophytes (green), using the WEGO (Web Gene Ontology Annotation Plot) tool

About 54% of the DETs up-regulated in embryos and 58% of the DETs up-regulated in megagametophytes could be GO annotated (Additional file 3: Figure S2A). Cellular and metabolic processes were the most dominant groups in the Biological Process category both in embryos and megagametophytes (Fig. 3). Furthermore, transcripts assigned to response to stimulus were over-represented in megagametophytes. In both embryos and megagametophytes, enriched GO terms in the Molecular Function category included catalytic and binding activities, and in the Cell Component category the subcategories cell and cell part were the most abundant.

**Fig. 4:**
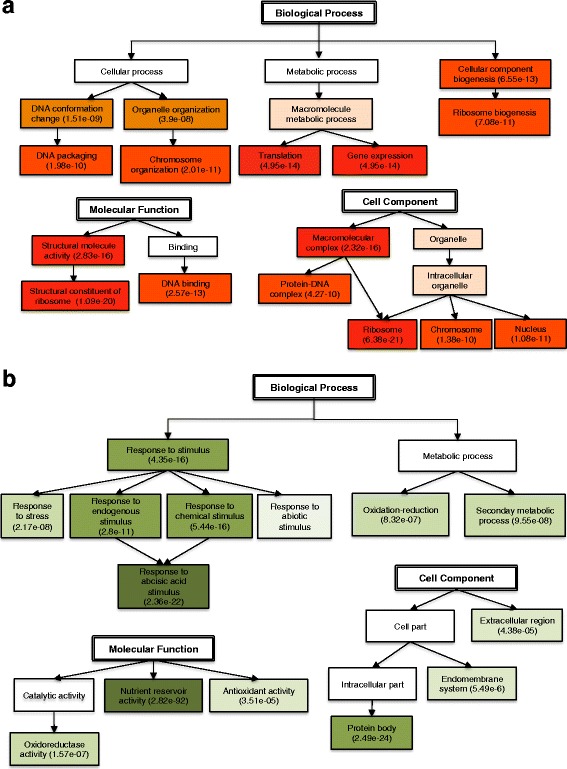
Summary of Gene Ontology (GO) enrichment analysis for differentially expressed transcripts during early seed development. The analysis included DETs with a fold change greater than 2 (FC > 2) identified in any of the pairwise comparisons between embryos and megagametophytes. The most abundant classes in each category, Biological Process, Molecular Function and Cell Component are shown for **a** embryos and **b** megagametophytes. Level of enrichment is proportional to color intensity. FDR values are presented in parenthesis. Detailed information is shown in Additional file 3: Figures S2 and S3

By increasing the GO annotation level, it was found that transcripts up-regulated in embryos were enriched for diverse Biological Processes such as cellular component biogenesis and cellular and metabolic processes related to chromosome organization, DNA packaging, translation and gene expression (Fig. 4a and Additional file 3: Figure S3). In the megagametophytes the up-regulated transcripts were highly enriched in response to stimulus, such as response to stress and to chemical and endogenous stimulus, including response to abscisic acid (ABA) (Fig. 4b and Additional file 3: Figure S4). In the Molecular Function category, assignments in the embryos were mainly related to DNA binding and structural constituent of ribosome. Both activities are highly related to gene expression and protein synthesis. In the megagametophytes, transcripts functioning in nutrient reservoir activity were highly over-represented (FDR = 2.82e-92). Transcripts identified in embryos for the Cell Component category showed enrichment for nucleus, ribosome and protein-DNA complex (Fig. 4a) and transcripts in megagametophytes were enriched mainly in protein body component (Fig. 4b). As expected, transcripts up-regulated in embryos showed GO enrichment for different cellular processes and functions in DNA-packaging, translation and gene expression. These processes are important during active cell proliferation [29]. Transcripts up-regulated in megagametophytes were enriched for accumulation of storage material and response to chemical and endogenous stimuli. This might indicate that the megagametophyte, in a similar way as the endosperm, can sense environmental signals and induce the corresponding signalling pathways for regulating embryo development [30].

We carried out pairwise comparisons between the group of transcripts showing the highest differences in abundance between embryos and megagametophytes at each developmental stage (Additional file 5). Transcripts related to members of the Arabidopsis cytochrome P450 gene family (*CYP78A7*, *CYP78A8* and *CYP71B22*) showed, at all developmental stages, high accumulation in embryos but low or no accumulation (RPKM close to 0) in megagametophytes. Up-regulated transcripts in megagametophytes were mainly related to the Arabidopsis 12S seed storage protein family (*CRB*, *CRC*, *CRD*), also known as cruciferins. These proteins are involved in nutrient reservoir activity and are the major sources of nitrogen and carbon during early seed germination [31]. The RPKM values of cruciferin-related transcripts were similar at all developmental stages. The majority of the transcripts up-regulated in the megagametophytes had no hits against the TAIR database (Additional file 5).

At stage E1, E2 and E3DO, transcripts related to genes encoding for cell wall modifications (expansins, cellulose metabolism, endoglucanase, pectin-acetylesterase and pectin-lyase) were detected. Specifically at stage E1, a putative homolog to *SOMATIC EMBRYOGENESIS RECEPTOR-LIKE KINASE1* (*SERK1*)*,* as well as transcripts related to genes involved in response to auxin and other hormones such as *INDOLE-3ACETATE O-METHYLTRANSFERASE 1* (*IAMT1*), *SKP1-LIKE PROTEIN 1A* (*SKP1A*), *GAMMA-VACUOLAR-PROCESSING ENZYME* (*GAMMA-VPE*)*, GIBBERELLIN-REGULATED PROTEIN 2* (*GASA2*) and *GLUTATHIONE S-TRANSFERASE U17* (*GSTU17*) were highly abundant (Additional file 5, Up in E1). Transcripts related to nucleosome assembly (histones) were detected at all developmental stages except at stage E1. Other transcripts up-regulated from stage 2 onwards coded for proteins related to stress responses i.e. non-specific *LIPID-TRANSFER PROTEIN 3* (*LTP3*)*, SUGAR TRANSPORT PROTEIN 13* (*STP13*) or *ABSCISIC ACID INSENSITIVE 4* (*ABI4*). Transcripts up-regulated at stage E4 included transcripts related to cell signalling, negative regulation of cell division and cell wall loosening, as well as transcripts related to development such as *PROTEIN RALF-like 34* (*RALFL34*), *FAMA, PLANTACYANIN* (*ARPN*) *or PECTIN ACETYLESTERASE* (*PAE9*) (Additional file 5, Up in E4).

In total 7704 TFs were detected during early seed development (Table 1). Out of these TFs, 2890 were differentially expressed with a fold change higher than two between embryos and megagametophytes (Additional file 6). The differentially expressed TFs belonged to 78 families, of which the bHLH, FAR1, TRAF and NAC families were the largest (Additional file 7: Figure S5 and Additional file 6, Family distribution). In general, the number of TFs belonging to each family was higher in embryos than in megagametophytes. Interestingly, some of the TF families were enriched differently in embryos and megagametophytes during seed development e.g. for bHLH, C3H, NAC, AP2-EREBP and TRAF (Additional file 7: Figure S6). In addition, sixteen TF families were detected only in embryos and four TF families were detected only in megagametophytes. In general, several TFs belonging to families specifically expressed in embryos were involved in plant growth and development, while TF families detected only in megagametophytes were related to responses to stress and other stimuli [32–34].

#### Differentially expressed transcripts during embryo development

**Fig. 5:**
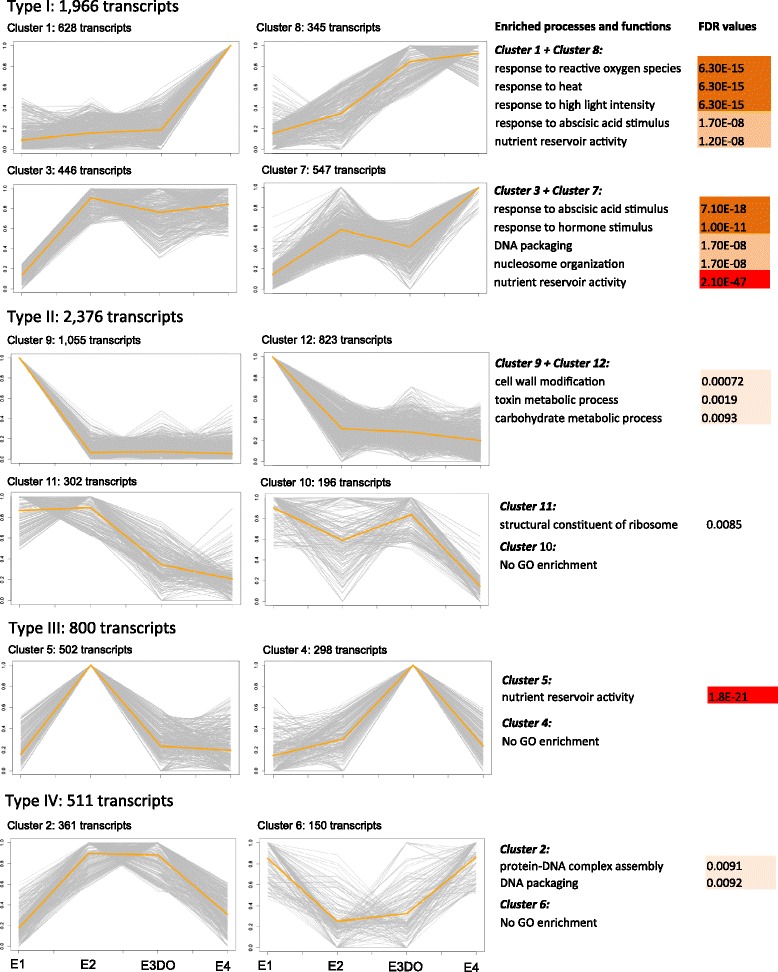
Clustering of differentially expressed transcripts identified in the pairwise comparisons between embryo developmental stages. The analysis included DETs with a FC > 2 and RPKM > 10. For each transcript, RPKM values were normalized to its maximum RPKM value during embryo development. Normalized values were subjected to k-means clustering method and classified into 12 different clusters, based on their expression level across the four developmental stages. The Y-and X-axis represent relative expression (from 0 to 1) and different embryo developmental stages, respectively. Enriched processes and functions and their enrichment level (FDR) are presented for each cluster. The level of enrichment is proportional to colour intensity

In total, 18,234 DETs with a fold change higher than two were identified in the pairwise comparisons between embryos at different developmental stages (Additional file 8: Figure S7). When including only transcripts with a RPKM > 10, 6669 DETs were detected. To provide an overview of the expression patterns of these DETs during embryo development, k-means clustering analysis was performed (DETs from subordinate embryos were excluded from this analysis). Four types of expression profiles were detected, where type I and II included four clusters each and type III and IV include two clusters (Fig. 5). The accumulation of transcripts belonging to type I increased throughout the course of embryo development. Transcripts in cluster 1 and 8 were specifically enriched for processes related to response to abiotic stress, and transcripts in cluster 3 and 7 were highly enriched for nutrient reservoir activity (FDR = 2.10e-47), response to ABA and other hormones. The expression of type II transcripts decreased during embryo development. However, the accumulation pattern differed among the four clusters. Transcripts in cluster 9 and 12 were abundant for cell wall modification, toxin and carbohydrate metabolic processes, while cluster 11 included a higher number of transcripts with a function in structural constituents of ribosomes. Type III transcripts showed high accumulation at only one intermediate developmental stage (E2 or E3DO). Transcripts within cluster 5, mainly accumulated at stage E2, were highly enriched for nutrient reservoir activity. However, no significant GO enrichment was obtained for cluster 4. The expression level of type IV transcripts was either high or low at both E2 and E3DO stages. Cluster 2 included transcripts involved in DNA packaging and protein-DNA complex assembly. Together the GO enrichment analyses of the clusters showed that the abundance of transcripts related to stress response and nutrient activity increased during embryo development, while the abundance of transcripts related to cell wall modification decreased.

**Fig. 6:**
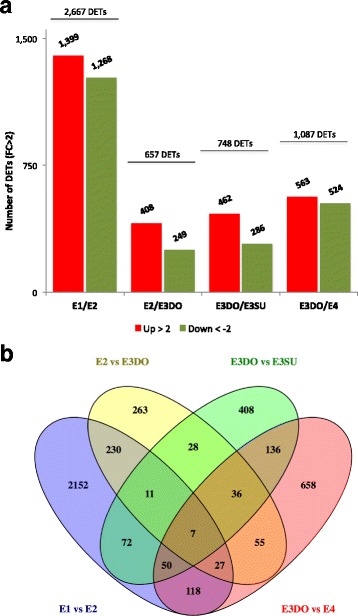
Differentially expressed transcripts (DETs) between consecutive stages during embryo development. The analysis included DETs with FC > 2 and RPKM > 10 identified in any of the four pairwise comparisons. **a** Histogram showing the number of up-regulated (red bars) and down-regulated (green bars) DETs between consecutive developmental stages. **b** Venn diagram showing the number of common and specific DETs between consecutive pairwise comparisons

When comparing embryos at consecutive developmental stages, including subordinate embryos, 4411 DETs were detected. The highest number of DETs (2667) was detected in the comparison between embryos at stage E1 and E2, and 80% (2152) of these DETs were only detected in this pairwise comparison (Fig. 6a and b). DETs highly accumulated at stage E1, were enriched for Biological Processes related to cell wall loosening, organization and modification, with a beta-expansin (*EXPB1*)-related transcript having the highest fold-change (Additional file 9, E1xE2 Up). Furthermore, 28 TFs involved in several developmental processes were detected, out of which transcripts related to *LOB DOMAIN-CONTAINING PROTEIN 29* (*LBD29*) and *SERK1,* as well as some members belonging to the homeobox-leucine zipper protein family (*HAT5* and *HB13*), showed a high fold-change (Additional file 8: Table S7 and Additional file 10, E1xE2 Up). Transcripts that were over-represented in E2 were enriched for processes related to response to ABA, hormone stimulus, nucleosome organization and nutrient reservoir activity (Additional file 9, E1xE2 Down). These DETs included 21 TFs that were GO annotated for developmental processes (Additional file 8: Table S7 and Additional file 10, E1xE2 Down).

Close to 660 DETs were identified when comparing embryos at stage E2 and E3DO (Fig. 6a). Transcripts assigned to response to ABA and hormone stimulus showed higher accumulation at stage E2, and those involved in response to abiotic stress were enriched at stage E3DO (Additional file 9, E2xE3DO). When comparing dominant embryos at stage E3DO and stage E4, 1087 DETs were detected (Fig. 6a). Transcripts up-regulated in E3DO embryos were mainly related to axis specification processes, while transcripts up-regulated at stage E4 were involved in processes related to response to hormone stimulus and lipid transport (*LTP3* and *LTP4*) (Additional file 9, E3DOxE4 Down). TFs, differentially expressed in embryos at stage E3DO and E4, which were annotated to developmental processes, included transcripts related to *AUXIN RESPONSIVE FACTOR 2* (*ARF2*), *LEUNIG* (*LUG*), *WUSCHEL-RELATED HOMEOBOX* (*WOX*), *CYP78A7* and *ARABIDOPSIS NAC DOMAIN CONTAINING PROTEIN 9* (*ANAC009*) (Additional file 8: Table S7 and Additional file 10).

By comparing dominant and subordinate embryos at stage E3, it was possible to detect 748 DETs (Fig. 6a). Many of the transcripts up-regulated in dominant embryos were related to carbohydrate metabolic processes and axis specification processes (Additional file 9, E3DOxE3SU Up). DETs enriched in subordinate embryos were involved in response to water stress (including water deprivation) and lipid transport. NAC and HB were the largest TF families in dominant embryos, while in subordinates MYB-related factors were the most abundant (Additional file 10, TF families).

**Fig. 7:**
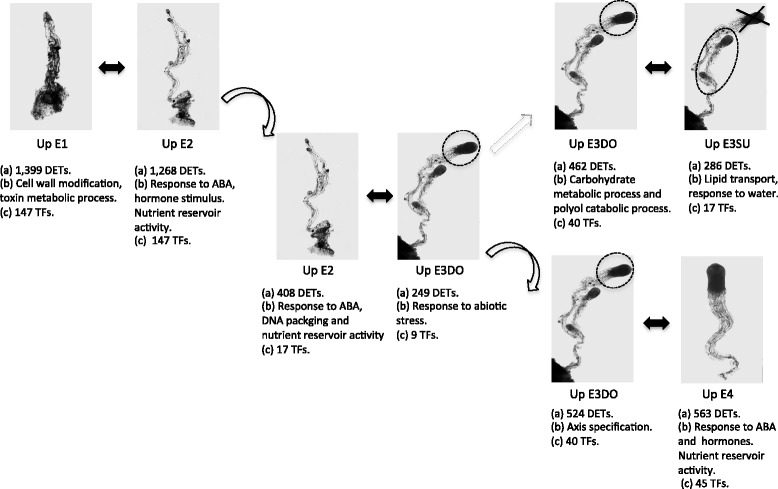
Schematic summary of the results obtained from the pairwise comparisons between consecutive stages during embryo development (also see Additional files 9 and 10). (*a*) Number of DETs detected; (*b*) GO enriched Biological Processes; (*c*) Number of TFs identified

A schematic summary of the results obtained from the pairwise comparisons between consecutive stages during embryo development is presented in Fig. 7. Together our results show that processes involved in cell-wall modifications, hormone signalling, axis specification and stress-induced responses are activated during early embryo development. A strict regulation of cell division, elongation and adhesion is critical during embryonic patterning formation. Auxin is perhaps the most pervasive signalling molecule in plants and has been implicated in many developmental processes including embryogenesis in both angiosperms and conifers [35–37]. In several studies it has been shown that genes related to stress are over-represented during early embryo development [12, 16, 17, 38]. Furthermore, many of the differentially expressed TFs that belong to the largest families (bHLH, FAR1, NAC and AP2-EREBP) are related to cellular and developmental processes, hormone signalling and stress responses [39–42].

#### Differentially expressed transcripts between different developmental stages in megagametophytes

DETs identified in the pairwise comparisons between megagametophytes at consecutive developmental stages were also subjected to k-means clustering, resulting in 12 different clusters grouped into five types of expression profiles (Additional file 11: Figure S8). No significant GO enrichment processes or functions (FDR < 0.05) were assigned to any of the clusters. The accumulation of type I transcripts increased from stage M1 to stage M4. Transcripts related to response to stimulus and regulation of biological process were the most abundant. The expression of type II transcripts decreased from stage M1 to stage M4. Type II clusters were abundant in transcripts with GO terms associated with cell wall organization, and reproductive and developmental processes. Type III transcripts accumulated either at stage M2 or stage M3. GO terms assigned to clusters in type III were mainly related to response to stimulus. Type IV and V included only one small cluster each. Cluster 6, with transcripts accumulating both at stages M2 and M3, presented a high percentage of DETs responding to stimulus, while transcripts from cluster 11 were annotated only for metabolic and cellular processes.

A total of 600 DETs (FC < 2, RPKM > 10) were detected in the pairwise comparisons between megagametophytes at consecutive developmental stages. No significant enriched processes or functions were found in any of the pairwise comparisons. Similar to embryos, the highest number of DETs was detected during the transition from stage M1 to M2, and 85% of the DETs were specifically detected in this pairwise comparison (Additional file 11: Figure S9A and B). The number of transcripts annotated for developmental processes decreased from 10 at stage M1 to 2 at stage M4. Meanwhile, the number of DETs with GO terms associated with response to stress and stimulus remained more constant (Additional file 11: Figure S9C). In addition, transcripts encoding for proteins belonging to the small Heat Shock Protein (sHSP) family, known for its role in stress response, showed similar accumulation during all developmental stages (Additional file 12, M1xM2 Down, M2xM3 Up).

Ten DETs with assigned GO terms related to development were specifically detected at stage M1, in which putative homologs to expansins (*EXPB1*s) and *AGAMOUS-like MADS-box* (*AGL11*) were included. *AGL11* was not detected in embryos at any developmental stage. In accordance, *AGL11* was expressed in the endosperm but not embryos of *Brachypodium distachyon* (Expression Atlas data from EMBL-EBI). Several transcripts related to metal ion transport, e.g. *Copper Transporter 5* (*COPT5*) and *Zinc Transporter 11* (*ZIP11*)*,* were detected at stage M1 (Additional file 12, M1xM2 Up). ZIP transporters participating in ion translocation during embryo and endosperm development have been detected in maize seeds [43]. At stage M4, a transcript related to *AtEP3,* encoding for an endochitinase, was highly abundant (Additional file 12, M3xM4 Down). A homologous gene, *Chia4-Pa1*, has been shown to be expressed in the single cell-layered zone surrounding the corrosion cavity in the megagametophyte in Norway spruce seeds [44].

### Expression of selected transcripts during early embryo development

**Fig. 8:**
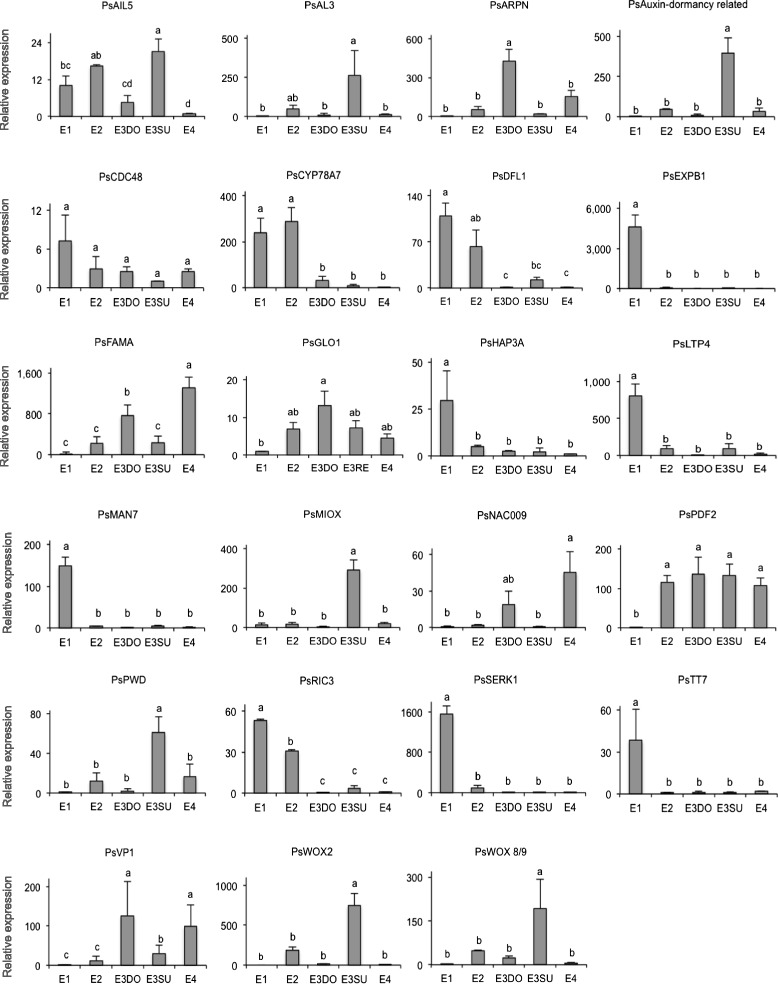
Quantitative real-time (qRT)-PCR analysis of the relative transcript level of selected genes during early embryo development. Relative expression levels, estimated by the Livak method (2-ΔΔCt), are referred to the developmental stage showing the minimum accumulation for each transcript. Expression values are normalized against two reference genes ELONGATION FACTOR-1 (EF1) and PHOSHOGLUCOMUTASE (PHOS). Transcript levels are means ± SD of three or four biological replicates, with two technical replicates each. Different letters indicate significant differences in the relative expression level (Student’s t-test, *P* < 0.05)

The transcript levels of selected DETs were tested by qRT-PCR in four biological replicates. The selection was based on the estimated expression (RPKM values) obtained from the transcriptome data and functional annotations of homologous genes in other species, mainly Arabidopsis, that have been related to embryo development. Gene sequence information in conifers is limited, thus for convenience, we refer each conifer transcript to the Arabidopsis gene that it shares most sequence similarity to. We have taken this approach for making it possible to get a general idea about which processes might be important during early embryo development. The results generated from qRT-PCR analysis are presented in Fig. 8.

Transcripts related to *ENDO-BETA-MANNASE 7* (*MAN7*), *TRANSPARENT TESTA7* (*TT7*), *EXPB1*, *SERK1*, *LTP4*, and *HAP3A* were highly abundant at stage E1 and decreased significantly at stage E2.

In Arabidopsis seeds, the mannanase-encoding gene, *AtMAN7,* is expressed in the micropylar endosperm and in the radicle tip just before radicle emergence [45]. We assume that the high expression of *PsMAN7* in E1 embryos might facilitate their penetration into the nutritious megagametophyte.

Early embryogenesis is a critical developmental phase when the apical-basal polarity is established through directional auxin transport mainly mediated by auxin influx and efflux carriers [46]. In addition, flavonols can act as negative regulators of auxin transport [47]. *AtTT7* encodes flavonoid 3′hydroxylase, a flavonol biosynthetic enzyme. Down-regulation of *PsTT7* from stage E2 might reflect that auxin transport is increased from the cleavage stage and during further embryo development. Previous studies have associated the action of expansins in cell wall loosening, expansion, dissemble or separation [48, 49]. A high expression of *PsEXPB1* at stage E1 might indicate the importance of loosening the cell walls to allow separation of the four early embryos. *AtSERK1* marks cells that are competent to form embryos, and it also influences the competence of the cells to differentiate into embryos [50]. The high expression of *PsSERK1* at stage E1 might be important for stimulating the four apical cells to differentiate into separate embryos and thereby stimulate the cleavage process to start. Directly after the first cleavage, the four new embryos should develop further. We assume that down-regulation of *PsSERK1* is important for blocking a second round of cleavage.

Another set of transcripts, represented by putative homologs to *CYP78A7*, *DWARF IN LIGHT 1* (*DFL1*) and *ROP-INTERACTIVE CRIB MOTIF-CONTAINING PROTEIN 3* (*RIC3*), were also highly abundant at stage E1 but declined successively during later developmental stages.

Cytochrome P450s are involved in the metabolism of most phytohormones and many secondary metabolites in plant cells. Overexpression of a member of the CYP78A family in rice (*Oryza sativa*) promotes cell proliferation but reduces the size of the embryos [51]. Furthermore, the gene product of *AtDFL1*, which is involved in auxin signal transduction, can inhibit cell elongation [52]. Before the development of the dominant embryo, the four early embryos are equal-sized. Although it is not known which mechanisms are restricting the growth of the embryonal mass of stage 2 embryos, our results indicate that PsCYP78A and PsDFL1 might be involved. *AtRIC3* is important for tip growth of pollen tubes [53]. Early embryos in *Pinus,* developing after the cleavage process, begin their development by apical cell growth [7]. A high expression of *PsRIC3* at stage E1 and E2 might reflect that these embryos develop by apical cell growth.

The level of transcripts related to *NAC009*, *FAMA*, *PROTODERMAL FACTOR2* (*PDF2*)*,* and *VIVIPAROUS1* (*VP1*) were low at stage E1 but increased during later stages. Putative homologs to *PLANTACYANIN* (*ARPN*) and *GLYOXALASE I* (*GLOI*) showed a higher accumulation at stage E3DO. In addition, a peak of transcript abundance in subordinate embryos was observed for transcripts related to *WOX2*, *WOX8/9*, *AINTEGUMENTA-like 5* (*PsAIL5), PHOSPHOGLUCAN WATER DIKINASE* (*PWD*), *MYO-INOSITOL OXYGENASE 1* (*MIOX1*), *ALFIN-like 3* (*AL3*) and a transcript encoding Auxin-dormancy-related protein.

*ANAC009* is expressed in root cap stem cells where it promotes periclinal cell divisions [54]. FAMA, a basic helix-loop-helix protein, regulates a critical switch between division and differentiation during stomatal development [55]. In Norway spruce, the apical-basal polarization during early embryogeny proceeds through the establishment of the meristematic cells of the embryonal mass and the terminally differentiated, expanding suspensor cells [56]. The high expression of *PsNAC009* and *PsFAMA* at stage E3DO and E4, but not at stage E3SU, may reflect the importance of correct cell division pattern for the development of dominant embryos. *PaWOX8/9* regulates the orientation of the cell division plane in the basal part of the embryonal mass during early and late embryogeny in Norway spruce [57]. In accordance, *PsWOX8/9* was expressed at all analysed developmental stages, however, the expression was significantly higher at stage E3SU. Although we do not know how overexpression of *PaWOX8/9* affects early embryo development, it is tempting to assume that the high expression of *PsWOX8/9* in subordinate embryos inhibits further development of the embryos or is only a consequence of a blocked development caused by other factors. In Arabidopsis plants overexpressing *AtARPN*, the endothecium degenerates, probably as a consequence of plantacyanin-induced precocious PCD [58]. The terminally differentiated suspensor cells in early embryos of Scots pine and Norway spruce are eliminated by PCD [9, 59]. The high expression of *PsARPN* in E3DO and E4 embryos, but not in E3SU, coincides with the degeneration of the suspensor cells. Taken together, these results suggest that the apical-basal polarization is strictly regulated in dominant embryos but not in subordinate embryos.

*Picea abies HOMEOBOX 1* (*PaHB1*), a homolog of *AtPDF2*, and *PaWOX2* are important for specification of the protoderm in somatic embryos of Norway spruce [60, 61]. Furthermore the expression of a Norway spruce *LTP* gene (*Pa18*) switches from a uniform expression in proembryogenic masses to a protoderm-specific localization in developing somatic embryos [62]. We assume that the differential expression of *PsLTP4* and *PsPDF2* is reflecting specification of the protoderm, which would indicate that radial patterning is regulated in a similar way in dominant and subordinate embryos. The expression pattern of *PsWOX2* was similar to that of *PsWOX8/9*, both transcripts were expressed at all developmental stages, but with significantly higher levels in subordinate embryos. This is probably related to the blocked development of the subordinate embryos.

We have previously shown that *PsHAP3A* is expressed during the morphogenic phase and *PsVP1* during the maturation phase [63]. *PsHAP3A* was down-regulated in both E3DO and E3SU embryos, while *PsVP1* was up-regulated in E3DO but not in E3SU embryos. Overexpression of the *Arabidopsis EMBRYOMAKER* (*AtEMK*), which is identical to *AtAIL5*, results in the formation of embryo-like structures on seedlings [64]. The authors concluded that *AtEMK* is a key player to maintain embryonic identity. The *PsAIL5* transcript was down-regulated in E3DO but not in E3SU embryos. Taken together, the low expression of *PsVP1* and high expression of *PsAIL5* in the subordinate embryos indicate that the transition from the morphogenic phase to the maturation phase is not completed in the subordinate embryos*.*

Although the functions of genes encoding for proteins related to PWD, MIOX1, AL3 and an Auxin-dormancy related protein during embryo development are not known, the fact that they are differentially expressed in E3DO and E3SU embryos indicates differences in metabolic processes between dominant and subordinate embryos.

Based on the processes identified in the GO enrichment analyses and the expression of the selected transcripts we suggest that processes related to embryogenic competence and cell wall loosening are involved in activating the cleavage process. Directly after cleavage, the growth of the embryos is restricted. Apical-basal polarization is strictly regulated in the dominant embryo, which has reached the maturation phase. However, functional studies must be performed before we will understand the processes controlling the successive development of the early embryos.

## Conclusion

In this study we have analysed changes in transcript accumulation during early seed development. GO enrichment analysis of transcripts differentially expressed in embryos and megagametophytes highlighted the importance of different cellular processes and functions related to DNA replication and translation in embryos and accumulation of storage material and transcripts responding to different stimuli in megagametophytes. Transcripts related to embryogenic competence, cell wall modifications, cell division pattern, axis specification, and response to hormones and stress factors were highly abundant and differentially expressed during embryo development. The abundance of representative DETs during different stages of embryo development was verified by qRT-PCR analyses.

However, it has to be kept in mind that we have had to focus on genes that have been characterized in angiosperms. Further functional studies of the differentially expressed genes, and at least those which have been annotated to angiosperm genes without known function or that completely lack an angiosperm homolog, will increase our understanding of the regulation of embryo development in *Pinus*.

## Additional files

Additional file 1:
**Table S1.** Number of 454 sequencing reads in the seed transcriptome. **Table S2.** Results from transcriptome assembly (trimmed by Seqclean). **Table S3.** Results from transcriptome integration. **Table S4.** Length of transcripts belonging to the seed transcriptome. **Figure S1.** Venn diagrams showing the number of common and unique transcripts and TFs with detectable expression (RPKM > 0), at different developmental stages. Venn diagrams showing the number of transcripts at each developmental stage in (A) embryos and (B) megagametophytes. Venn diagrams showing the number of TFs at each developmental stage in (C) embryos and (D) megagametophytes. (PDF 393 kb)
Additional file 2:
**Table S5.** List of transcripts selected for qRT-PCR analyses. **Table S6.** Validation of the RNA-seq expression data. (PDF 88.2 kb)
Additional file 3:
**Figure S2.** Number of differentially expressed transcripts (DETs) with a fold-change greater than 2 (FC > 2) identified in each pairwise comparison between embryos and megagametophytes. (A) Total number of up-regulated transcripts in embryos and megagametophytes (B) Number of up-regulated transcripts in embryos (orange) and megagametophytes (green) at different developmental stages during early seed development. **Figure S3.** GO enrichment analysis of up-regulated transcripts (FC > 2) in embryos, identified in any of the pairwise comparisons between embryos and megagametophytes at the four developmental stages shown in Fig. 1. Enrichment in Biological Processes was obtained using AgriGO Toolkit database (FDR < 0.05). High significant levels are represented by red squares. **Figure S4.** GO enrichment analysis of up-regulated transcripts (FC > 2) in megagametophytes, identified in any of the pairwise comparisons between embryos and megagametophytes at the four developmental stages shown in Fig. 1. Biological Process enrichment was obtained using AgriGO Toolkit database (FDR < 0.05). High significant levels are represented by red squares. (PDF 672 kb)
Additional file 4: Differentially expressed transcripts, with a fold change higher than 2, in at least one of the pairwise comparisons between embryos and megagametophytes. This file includes a list of all the up-regulated transcripts in embryos and megagametophytes respectively, resulting from the pairwise comparisons E1 vs M1, E2 vs M2, E3DO vs M3, E3SU vs M3 and E4 vs M4. Transcript name (Transcript ID) and the best match against TAIR database (At locus ID, At name and At annotation) are shown. (XLSX 626 kb)
Additional file 5: Differentially expressed transcripts in embryos and megagametophytes at different stages of seed development. DETs with the highest fold-changes in embryos and megagametophytes at different developmental stages are included. Transcript name (Transcript ID), fold-change (log_2_ E/M), expression values (RPKM Embryos and RPKM Megagametophytes) and the best match against TAIR database (At locus ID, At name, At annotation) are shown. (XLSX 6.02 kb)
Additional file 6: Transcription factors (TFs) differentially expressed, with a fold-change higher that 2, in any of the pairwise comparison between embryos and megagametophytes. A list of differentially expressed TFs (DETFs) in embryos and megagametophytes respectively, resulting from the pairwise comparisons E1 vs M1, E2 vs M2, E3DO vs M3, E3SU vs M3 and E4 vs M4 is included in this file. TF name (Transcript ID), TF family, and the best match against TAIR database (At locus ID, At name and At annotation) are indicated. The number of all DETFs detected for each family during seed development in embryos and megagametophytes is also included. (XLSX 200 kb)
Additional file 7:
**Figure S5.** Distribution of up-regulated TF family members in embryos and megagametophytes. Presented data are based on TF family members differentially accumulated (FC > 2) during seed development in any of the pairwise comparisons between embryos and megagametophytes. Orange bars show the number of TFs belonging to each family in embryos and green bars in megagametophytes. TFs were classified into TF families by using the publicly available PlantTFDB v 3.0 database. **Figure S6.** Abundance of the ten largest TF families differentially expressed between embryos and megagametophytes during seed development shown in Fig. 5. Number of members in each TF family detected at different developmental stages in (A) embryos and (B) megagametophytes. Subordinate embryos were excluded from this analysis. (PDF 312 kb)
Additional file 8:
**Figure S7.** Number of differentially expressed transcripts (DETs) with a fold-change greater than 2 (FC > 2) identified in each pairwise comparison between embryo developmental stages. Up-regulated and down-regulated transcripts are shown in red and green bars, respectively. The number of total DETs in each pairwise comparison is shown below. **Table S7.** Differentially expressed TFs (FC > 2), related to developmental process, detected in the pairwise comparisons between consecutive stages during embryo development. (PDF 341 kb)
Additional file 9: Differentially expressed transcripts (FC > 2; RPKM > 10) between consecutive developmental stages during embryo development. Only DETs with positive matches against TAIR database have been included. Transcript name (Transcript ID), fold-change (FC), expression values at each embryo developmental stage (RPKM) and the best match against TAIR database (At locus ID, At name, At annotation) are shown. GO enrichment analysis for the up- or down-regulated transcript list resulting from each pairwise comparison is also presented. For each GO enrichment result is shown the GO term, Onthology, (P = Biological Process, F = Molecular Function, C = Cell Component), Number of GO annotated transcripts belonging to each category among the up- or down-regulated transcript list (Number in input list), Number of total GO annotated transcripts belonging to each category in the seed transcriptome (Number in BG/Ref), p-value and level of enrichment (FDR). (XLSX 35.8 kb)
Additional file 10: Differentially expressed transcription factors (FC > 2; RPKM > 10), between consecutive developmental stages during embryo development. Up- and down-regulated TFs from each pairwise comparison, as well as the list and frequency of the TF families detected, are presented in this file. Transcript name (Transcript ID), fold-change (FC), expression values at each embryo developmental stage (RPKM), TF family and the best match against TAIR database (At locus ID, At name, At annotation) are indicated. TFs GO annotated for developmental biological process are coloured in red in the up-regulated TF lists and in green in the down-regulated TF list for each comparison. (XLSX 131 kb)
Additional file 11:
**Figure S8.** Clustering of differentially expressed transcripts identified in the pairwise comparisons between different megagametophyte developmental stages. The analysis included DETs with a FC > 2 and RPKM > 10. For each transcript, RPKM values were normalized to its maximum RPKM value during development. Normalized values were subjected to k-means clustering method and classified into different clusters, based on their expression levels across the four developmental stages. The Y-axis of the cluster chart represents relative expression (from 0 to 1). The percentage of transcripts with GO terms assigned to different Biological Processes (GO at level 2) for each type is shown. **Figure S9.** Differentially expressed transcripts between consecutive stages in megagametophytes during early seed development. The analysis included DETs, with FC > 2 and RPKM > 10, identified in any of the three pairwise comparisons. (A) Histogram showing the number of up- and down-regulated DETs between different developmental stages. (B) Venn diagram showing the common and specific number of DETs detected in the consecutive pairwise analysis. (C) Summary table showing the number of DETs (excluding those with no assigned matches against TAIR) involved in different processes in each development transition. (PDF 1.23 kb)
Additional file 12: Differentially expressed transcripts (FC > 2; RPKM > 10) between consecutive megagametophyte developmental stages. Only DETs with positive matches against TAIR database are included. Transcript name (Transcript ID), fold-change (FC), expression values at each megagametophyte developmental stage (RPKM) and the best match against TAIR database (At locus ID, At name, At annotation) are shown. Transcripts GO annotated for developmental biological process are coloured in red or green, in the up- or down-regulated lists, respectively. All transcripts GO annotated for response to stimulus and stress are highlighted in bold. (PDF 12.2 kb)

## Abbreviations

DET: Differentially expressed transcripts
EST: Expressed sequence tag
FC: Fold-change
FDR: False discovery rate
GO: Gene ontology
ORF: Open reading frame
PCD: Programmed cell death
PlantTFDB: Plant transcription factor database
PUT: Putative sequences
qRT-PCR: Quantitative reverse transcription polymerase chain reaction
RPKM: Reads per kilobase per million mapped reads
TAIR: The Arabidopsis information resource
TF: Transcription factor
