# Supplementary material for: Transcript profiling for early stages during embryo development in Scots pine
Source: BMC Plant Biol. 2016 Nov 18;16:255. doi: 10.1186/s12870-016-0939-5 (PMC5116219; doi:10.1186/s12870-016-0939-5)

Table S1. Number of 454 sequencing reads in the seed transcriptome. Embryos and megagametophytes were sampled at the developmental stages shown in Figure 1.

Sample	# Raw Reads	# Reads for Assembly	# Bases for Assembly	% Reads for Assembly
E1	603,508	539,840	250,762,894	89.45%
E2	625,195	573,611	279,768,670	91.75%
E3SU	758,018	725,345	512,694,678	95.69%
E3DO	817722	780,954	552,917,241	95.50%
E4	749,430	711,685	506,198,526	94.96%
M1	745,590	708,247	499,030,847	94.99%
M2	800,707	768,722	521,997,230	96.01%
M3	789,571	754,048	516,959,353	95.50%
M4	712,754	671,451	465,102,991	94.21%
**Total**	**6,602,495**	**6,233,903**	**4,105,432,430**	**94.42%**

Table S2. Results from transcriptome assembly (trimmed by Seqclean).

	P. sylvestris
Isogroups	76,425
Isotigs	117,551
N50*	1,542
Max length	18,100
Min length	150
Mean length	1,242
Median length	977

Table S3. Results from transcriptome integration.

Source	Name tag	# Transcript (trimmed)	# Transcripts in final set
Newbler	contig / isotig	117,551	66,129
PlantGDB	PUT	67,744	54,759
EST	Cl / 118	2,161	1,050
**Total**		**187,456**	**121,938**

Table S4. Length of transcripts belonging to the seed transcriptome.

	**150-500 bp**	**500-1000 bp**	**1 kbp-2 kbp**	**>2 kbp**
N° transcripts	62,397	31,336	18,934	9,271
% from total transcripts	51.2 %	25.7 %	15.5 %	7.6 %

Figure S1. Venn diagrams showing the number of common and unique transcripts and TFs with detectable expression (RPKM>0), at different developmental stages. Venn diagrams showing the number of transcripts at each developmental stage in (A) embryos and (B) megagametophytes. Venn diagrams showing the number of TFs at each developmental stage in (C) embryos and (D) megagametophytes.

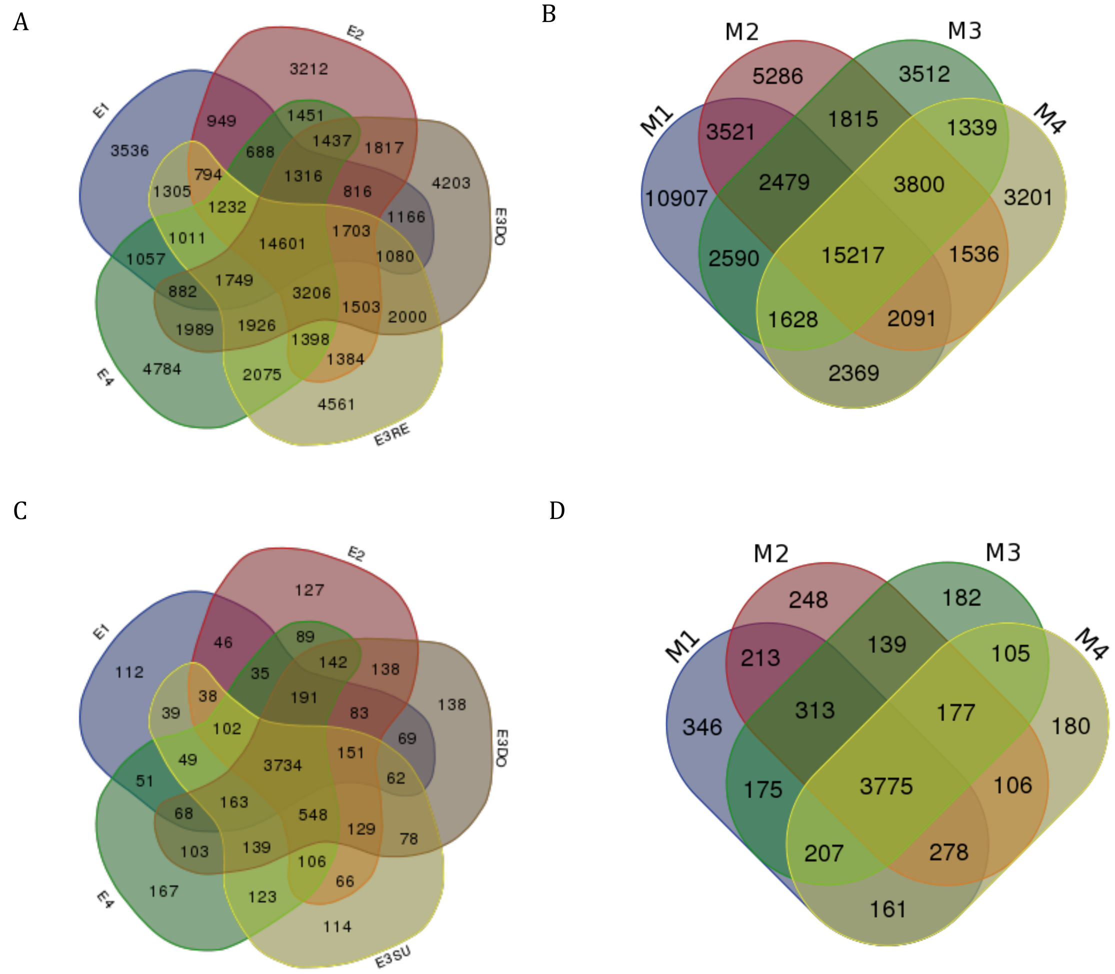

Supplement: Additional file 1: — Table S1. Number of 454 sequencing reads in the seed transcriptome. Table S2. Results from transcriptome assembly (trimmed by Seqclean). Table S3. Results from transcriptome integration. Table S4. Length of transcripts belonging to the seed transcriptome. Figure S1. Venn diagrams showing the number of common and unique transcripts and TFs with detectable expression (RPKM > 0), at different developmental stages. Venn diagrams showing the number of transcripts at each developmental stage in (A) embryos and (B) megagametophytes. Venn diagrams showing the number of TFs at each developmental stage in (C) embryos and (D) megagametophytes. (PDF 393 kb) [file 12870_2016_939_MOESM1_ESM.pdf]
