# Supplementary material for: Transcript profiling for early stages during embryo development in Scots pine
Source: BMC Plant Biol. 2016 Nov 18;16:255. doi: 10.1186/s12870-016-0939-5 (PMC5116219; doi:10.1186/s12870-016-0939-5)
Supplement: Additional file 2: — Table S5. List of transcripts selected for qRT-PCR analyses. Table S6. Validation of the RNA-seq expression data. (PDF 88.2 kb) [file 12870_2016_939_MOESM2_ESM.pdf]

Table S5. List of transcripts selected for qRT-PCR analyses.

Transcript ID	At locus ID(a)	At Gene name	Forward primer (5' è3')	Reverse primer (5' è3')
isotig37804	AT4G09960.3	AGL11	GCATTCCAAACCCTAGTAATCGACATTC	GCAAACTCGTAAATCTTGCCGTTATC
isotig56973	AT5G57390.1	AIL5	AATGGGGGCCAATCCATCTG	AGGGCTTTGCAGAAGCTGAT
isotig35435	AT3G42790.1	AL3	GGCATGGGTACCTACATCTCG	ACCACAAGCTCCACAAAGTGT
isotig87667	AT2G02850.1	ARPN	GGCAGTGACGCGATTACTCT	ATTTTCATCCCAGCCGAGCA
isotig10842	AT1G56220.3	Auxin-dormancy	CCTTGCCCTGATACTGAATTAGCC	ACACTAGCTCTGCGTTCTTTCAATTC
isotig22130	AT3G09840.1	CDC48	GATGATAACTCTGTGGTCTGTCTGC	GCACAACCTTGTTCATTCTAATCCTGG
isotig87231	AT5G20650.1	COPT5	TCCGTACAATTCCATGGAATCAGC	TGCATTCACTCCAAACAGCAG
isotig04574	AT5G09970.1	CYP78A7	GGCTCTATGTCCTCCTCTTTGGA	GCCAAAACAGCAGCGATGAGC
isotig21547	AT5G54510.1	DFL1	GAAGAAATACGGGCTCAACGGA	GTGTCTCCGTTAGCAATACGATGA
isotig19259	AT2G20750.1	EXPB1	GCGGATATAAGAGTCTAGTGAAACGG	CTTCACCTGATAGCAGGCTCC
isotig37162	AT3G24140.1	FAMA	ATTTTCCAGCAGAGAGAACAGCAGG	GTTCAGAAGTTGTTGAAAGCCATGC
isotig70889	AT5G48480.1	GLO1	TGCGGACGCCATAAGTTTCT	CGTGAAGGATGAGAGGGAGC
isotig35113	AT5G47670.1	HAP3A	AGCTCGATCTTCGGTCTTCA	TGCCAACTTCGGACATCATA
isotig04840	AT5G59720.1	HSP18.1	ATGGCCGATTTTTTGTATTTCGAGC	TCTTGCGTTCACCACTGATCT
isotig75537	AT4G10250.1	HSP22	TGAGGAAGGATGAAATCAAGATCG	GAAGCCTGAATTGCCTCCAGA
isotig01362	AT5G59310.1	LTP4	TATGTTACGGGGAGCGATGC	GGCTTGCCTGTCTGGAGTAG
isotig61465	AT5G66460.1	MAN7	GGAAGGATTCTATGGCGACTCAAC	CATCTGATCCAGCCAGCCAAG
isotig64994	AT1G14520.1	MIOX1	ACGAACTCGACCAT CGCAG	CTGACCAAAGGCATTGCTGT
isotig63158	AT1G26870.1	NAC009	ACAAGAATCTGCCTCCTAATGATGC	GCCCGAGACCATGTCTGAAT
isotig24154	AT4G11650.1	OSM34	GATGCAACAGCGCCTGTGTA	TGAACGTGCTTGAAGTATCATCC
contig03289	AT4G04890.1	PDF2	AAGTTAGCTGAGCGCATGGT	AAGGCCAACATTTGCACGAG
isotig87399	AT5G26570.1	PWD	TAGGGCTTGTAGGCCTTCCT	GTGCGTTGGGTAAGCACAAA
isotig85219	AT5G66400.1	RAB18	TCCGCTCCGCGTTCTGTCAA	TCCTGTTGTTCTTCCGCCATCT
isotig50515	AT1G05260.1	RIC3	GCTGACATTCTCGCTCTGGT	GATATCCTTCCGTCCCTGCGA
isotig48941	AT1G71830.1	SERK1	TGCATCCTTGCAGTCCAAACC	CTTCCCAGCTTGCTAAAACTTTATCC
isotig43729	AT5G07990.1	TT7	GAAACGGTCATCGGGGAAACG	GTATCCGTTCCAGCAGCCAA
isotig39945	AT3G24650.1	VP1	GTGCCCAATTTGGTTAATGG	GTGCCATCAAAGACAGCAGA
isotig25191	AT5G59340.1	WOX2	GCCACGAACACTAGGTGGGTT	CCCGATTTCTGCCGTGTAGGA
isotig37356	AT2G33880.1	WOX8/9	TACACGCAGCAGGACAACCAGT	TCGTTATTGCTTTCGTGTCCTGG
isotig23881	AT1G07920.1	EF1	CACCTTGGGAGTGAAGCAAATG	CGGAGTAGTGGCATCCATCTTG

(a) Best hit against TAIR database. See Material and Methods.

Table S6. Validation of the RNA-seq expression data. The Pearson correlation coefficient between the expression profiles obtained by RNA-seq and qRT-PCR was calculated separately for 30 transcripts (23 transcripts in embryos and 7 transcripts in megagametophytes). A coefficient of +1 indicates a perfect positive correlation, meanwhile -1 indicates a perfect negative correlation. A value close to zero indicates that no correlation exists between the profile patterns obtained by RNA-seq and qRT-PCR.

**Transcript**	**Correlation coefficient**
AIL5[a]	0.14
AL3 [a]	0.85
ARPN [a]	0.89
Auxin-dormancy related [a]	-0.83
CDC48 [a]	0.32
CYP78A7 [a]	-0.12
DFL1 [a]	0.93
EXPB1 [a]	1.00
FAMA [a]	0.99
GLO1 [a]	0.86
HAP3A [a]	0.89
LTP4 [a]	0.93
MAN7 [a]	1.00
MIOX [a]	-0.46
NAC009 [a]	0.95
PDF2 [a]	0.88
PWD [a]	0.95
RIC3 [a]	0.99
SERK1 [a]	1.00
TT7 [a]	0.99
VIP1 [a]	0.91
WOX2 [a]	0.56
WOX8/9 [a]	0.90
EXPB1[b]	1.00
AGL11 [b]	0.84
COPT5 [b]	1.00
HSP18.1 [b]	0.86
HSP22 [b]	0.97
OSM34 [b]	0.89
RAB18 [b]	0.44

[a] transcripts tested in embryos

[b] transcripts tested in megagametophytes
