# Supplementary material for: Transcript profiling for early stages during embryo development in Scots pine
Source: BMC Plant Biol. 2016 Nov 18;16:255. doi: 10.1186/s12870-016-0939-5 (PMC5116219; doi:10.1186/s12870-016-0939-5)

Figure S2. **Number of differentially expressed transcripts (DETs) with a fold-change greater than 2 (FC>2) identified in each pairwise comparison between embryos and megagametophytes**. (A) Total number of up-regulated transcripts in embryos and megagametophytes (B) Number of up-regulated transcripts in embryos (orange) and megagametophytes (green) at different developmental stages during early seed development.

A

Tissue	DETs	DETs with GO annotation	TF DETs
Embryos	12,906	6,986	2,091
Megagametophytes	5,732	3,334	799
Total DETs	18,638	10,320	2,890

B

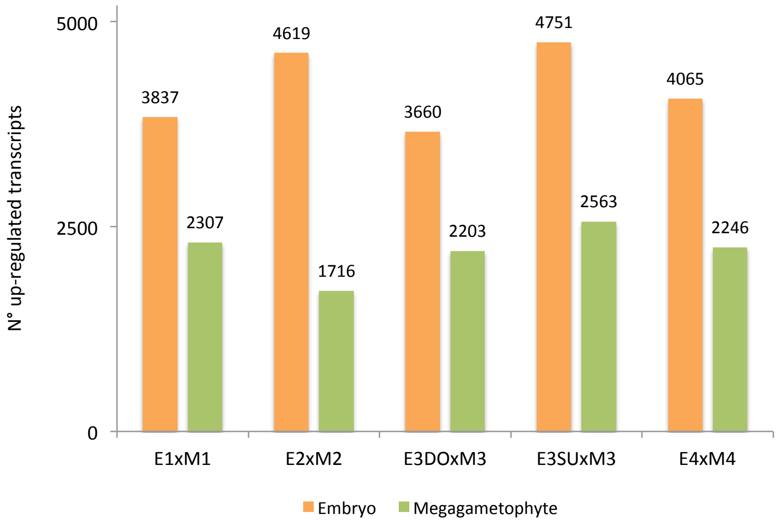

Figure S3. **GO enrichment analysis of up-regulated transcripts (FC>2) in embryos, identified in any of the pairwise comparisons between embryos and megagametophytes at the four developmental stages shown in Figure 1**. Enrichment in Biological Processes was obtained using AgriGO Toolkit database (FDR < 0.05). High significant levels are represented by red squares.

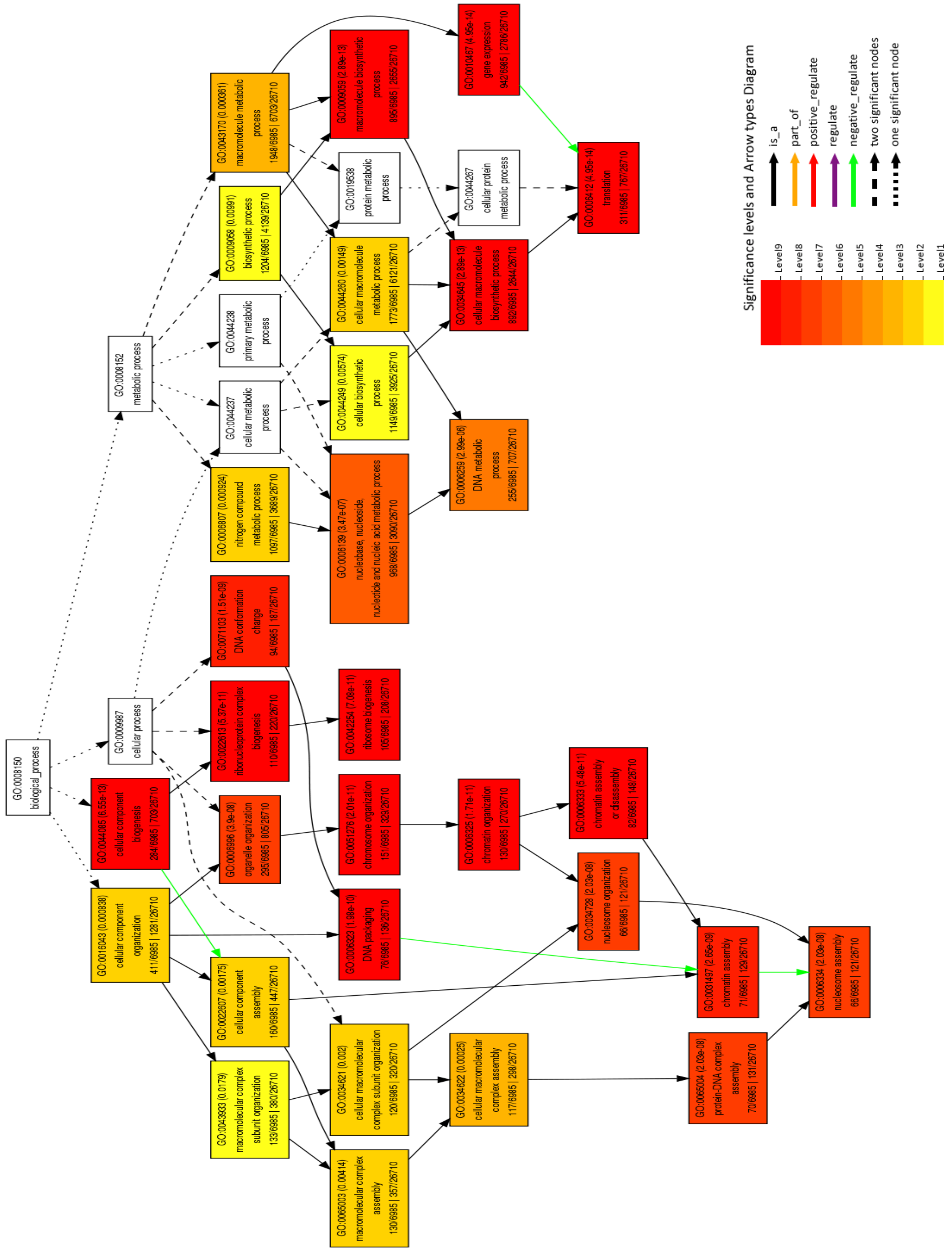

Figure S4. GO enrichment analysis of up-regulated transcripts (FC>2) in megagametophytes, identified in any of the pairwise comparisons between embryos and megagametophytes at the four developmental stages shown in Figure 1. Biological process enrichment was obtained using AgriGO Toolkit database (FDR < 0.05). High significant levels are represented by red squares.

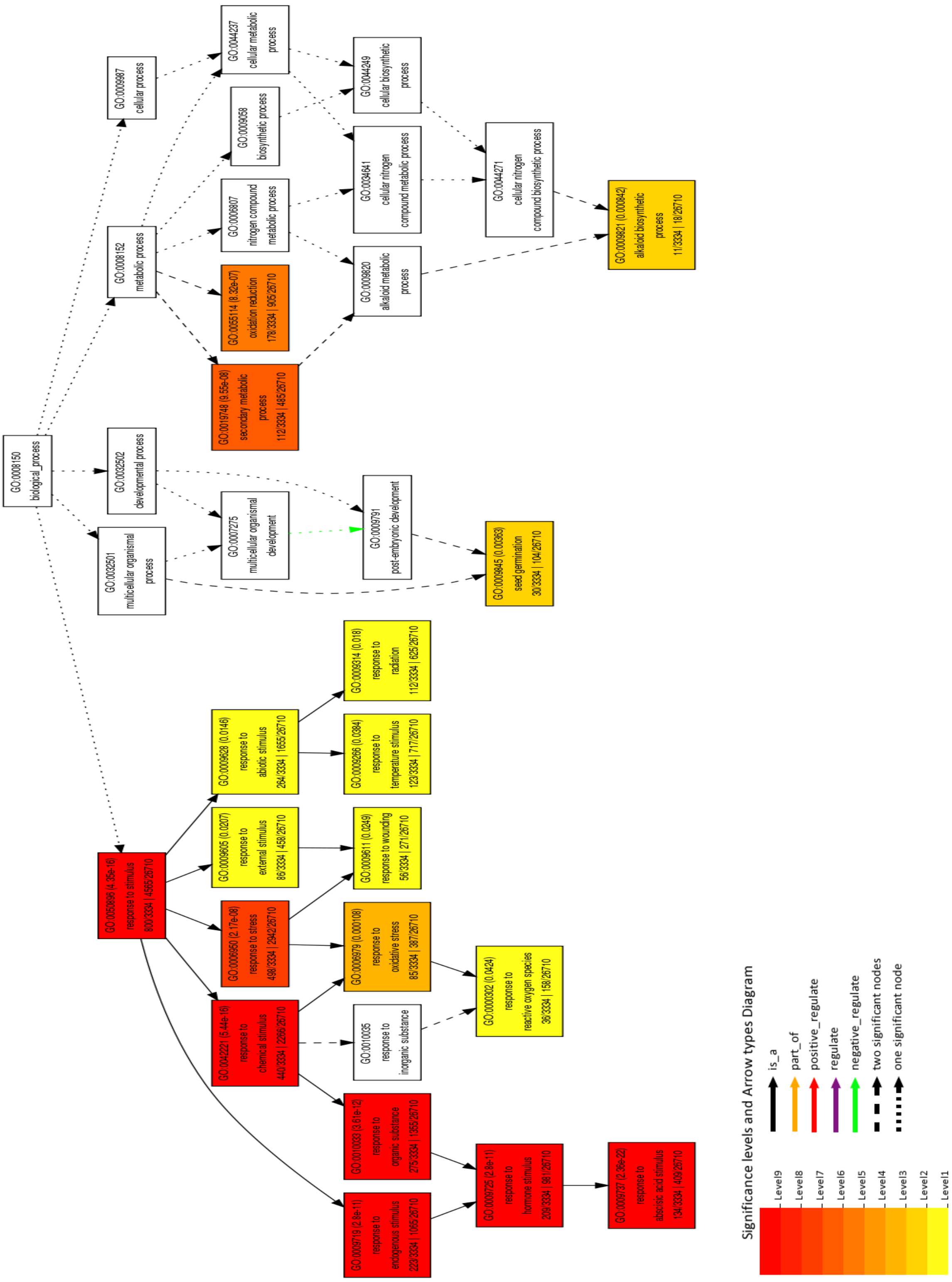

Supplement: Additional file 3: — Figure S2. Number of differentially expressed transcripts (DETs) with a fold-change greater than 2 (FC > 2) identified in each pairwise comparison between embryos and megagametophytes. (A) Total number of up-regulated transcripts in embryos and megagametophytes (B) Number of up-regulated transcripts in embryos (orange) and megagametophytes (green) at different developmental stages during early seed development. Figure S3. GO enrichment analysis of up-regulated transcripts (FC > 2) in embryos, identified in any of the pairwise comparisons between embryos and megagametophytes at the four developmental stages shown in Fig. 1. Enrichment in Biological Processes was obtained using AgriGO Toolkit database (FDR < 0.05). High significant levels are represented by red squares. Figure S4. GO enrichment analysis of up-regulated transcripts (FC > 2) in megagametophytes, identified in any of the pairwise comparisons between embryos and megagametophytes at the four developmental stages shown in Fig. 1. Biological Process enrichment was obtained using AgriGO Toolkit database (FDR < 0.05). High significant levels are represented by red squares. (PDF 672 kb) [file 12870_2016_939_MOESM3_ESM.pdf]
