# Supplementary material for: Transcript profiling for early stages during embryo development in Scots pine
Source: BMC Plant Biol. 2016 Nov 18;16:255. doi: 10.1186/s12870-016-0939-5 (PMC5116219; doi:10.1186/s12870-016-0939-5)

Figure S5. Distribution of up-regulated TF family members in embryos and megagametophytes. Presented data are based on TF family members differentially accumulated (FC>2) during seed development in any of the pairwise comparisons between embryos and megagametophytes. Orange bars show the number of TFs belonging to each family in embryos and green bars in megagametophytes. TFs were classified into TF families by using the publicly available PlantTFDB v 3.0 database.

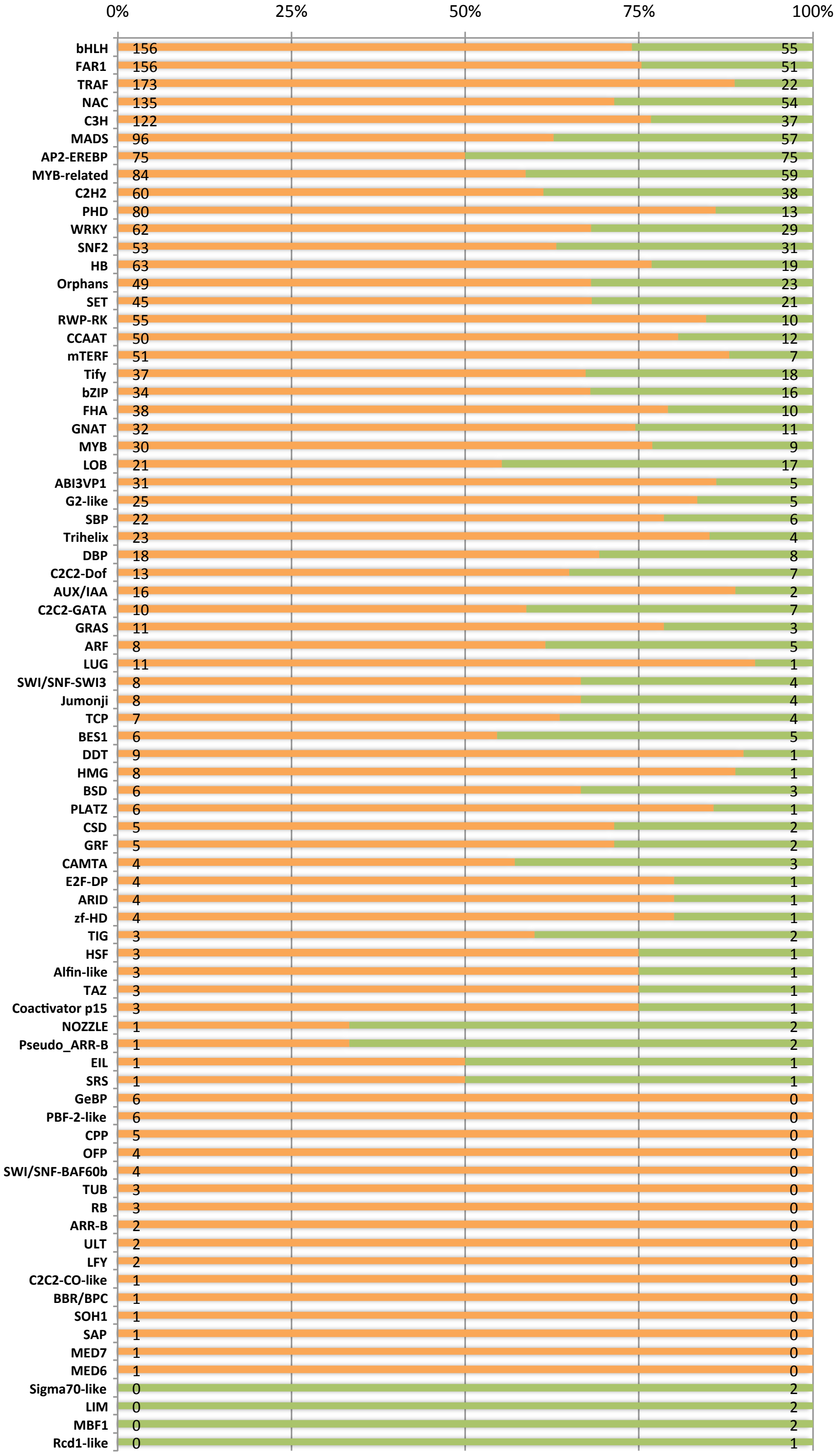

Figure S6. Abundance of the ten largest TF families differentially expressed between embryos and megagametophytes during seed development shown in Figure 5. Number of members in each TF family detected at different developmental stages in (A) embryos and (B) megagametophytes. Subordinate embryos were excluded from this analysis.

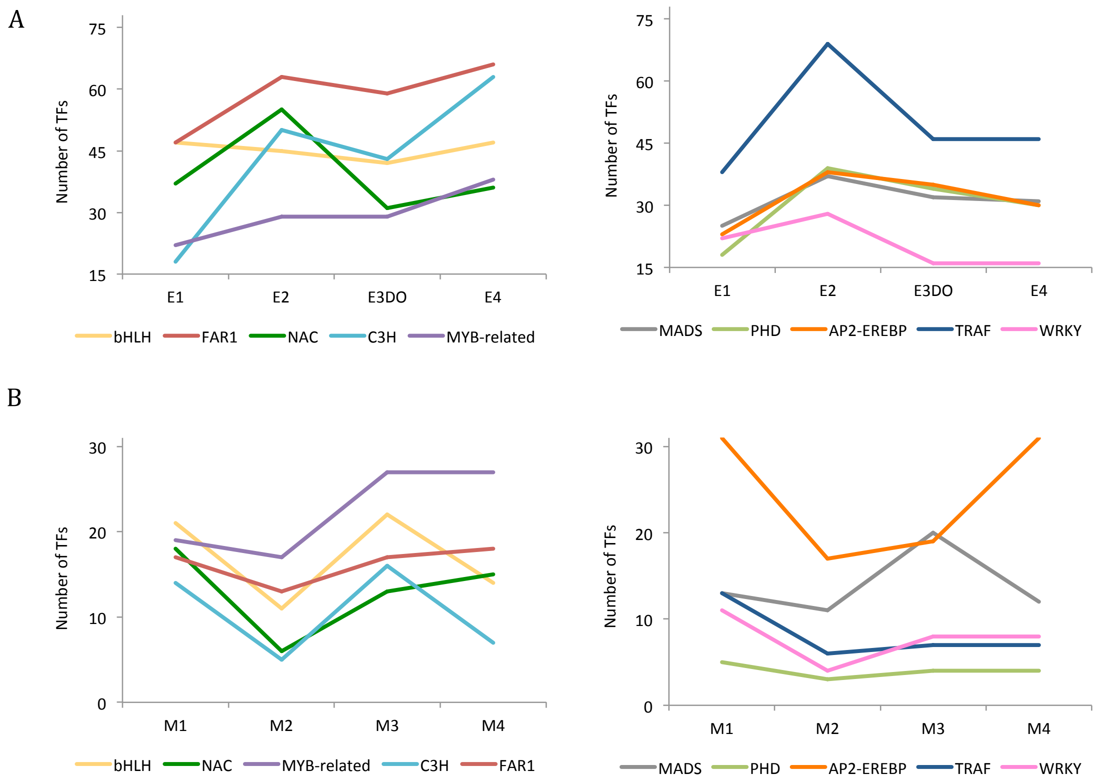

Supplement: Additional file 7: — Figure S5. Distribution of up-regulated TF family members in embryos and megagametophytes. Presented data are based on TF family members differentially accumulated (FC > 2) during seed development in any of the pairwise comparisons between embryos and megagametophytes. Orange bars show the number of TFs belonging to each family in embryos and green bars in megagametophytes. TFs were classified into TF families by using the publicly available PlantTFDB v 3.0 database. Figure S6. Abundance of the ten largest TF families differentially expressed between embryos and megagametophytes during seed development shown in Fig. 5. Number of members in each TF family detected at different developmental stages in (A) embryos and (B) megagametophytes. Subordinate embryos were excluded from this analysis. (PDF 312 kb) [file 12870_2016_939_MOESM7_ESM.pdf]
