# Supplementary material for: Transcript profiling for early stages during embryo development in Scots pine
Source: BMC Plant Biol. 2016 Nov 18;16:255. doi: 10.1186/s12870-016-0939-5 (PMC5116219; doi:10.1186/s12870-016-0939-5)
Supplement: Additional file 8: — Figure S7. Number of differentially expressed transcripts (DETs) with a fold-change greater than 2 (FC > 2) identified in each pairwise comparison between embryo developmental stages. Up-regulated and down-regulated transcripts are shown in red and green bars, respectively. The number of total DETs in each pairwise comparison is shown below. Table S7. Differentially expressed TFs (FC > 2), related to developmental process, detected in the pairwise comparisons between consecutive stages during embryo development. (PDF 341 kb) [file 12870_2016_939_MOESM8_ESM.pdf]

Figure S7. Number of differentially expressed transcripts (DETs) with a fold-change greater than 2 (FC>2) identified in each pairwise comparison between embryo developmental stages. Up-regulated and down-regulated transcripts are shown in red and green bars, respectively. The number of total DETs in each pairwise comparison is shown below.

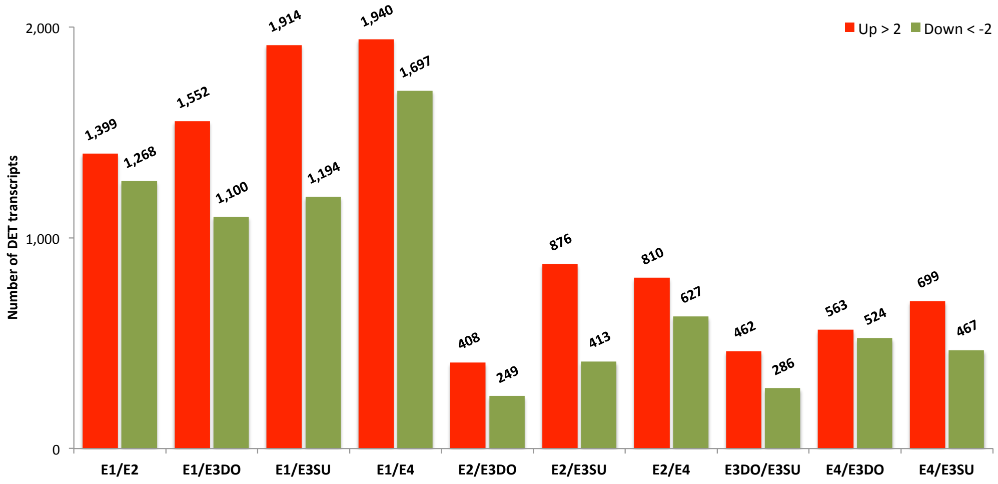

	E1/E2	E1/E3DO	E1/E3SU	E1/E4	E2/E3DO	E2/E3SU	E2/E4	E3DO/E3SU	E3DO/E4	E3SU/E4
**Total DETs**	2,667	2,652	3,108	3,637	657	1,289	1,437	748	1,087	1,166

Table S7. Differentially expressed TFs (FC>2), related to developmental process, detected in the pairwise comparisons between consecutive stages during embryo development. Positive values for fold-changes (FC) in each comparison indicate up-regulated transcripts, negative values indicate down-regulated transcripts.

Transcript ID	FC	At locus	At name	At annotation
**Pairwise comparison E1/E2**				
isotig68230	99	AT3G01470.1	HAT5	Homeobox-leucine zipper protein HAT5
isotig72228	99	AT3G58190.1	LBD29	LOB domain-containing protein 29
isotig95662	99	AT2G27230.1	LHW	Transcription factor LHW
isotig90693	99	AT3G54560.1	H2AV	Histone H2A variant 1
isotig48941	5.05	AT1G71830.1	SERK1	Somatic embryogenesis receptor kinase 1
isotig58389	4.86	AT5G07180.1	ERL2	LRR receptor-like serine/threonine-protein kinase ERL2
isotig44842	3.63	AT5G12330.2	LRP1	Protein LATERAL ROOT PRIMORDIUM 1
isotig39138	3.57	AT5G39660.1	CDF2	Cyclic dof factor 2
isotig59598	3.52	AT1G69780.1	ATHB-13	Homeobox-leucine zipper protein ATHB-13
isotig72491	3.29	AT3G58850.1	PAR2	Transcription factor PAR2
isotig61680	3.26	AT3G01470.1	HAT5	Homeobox-leucine zipper protein HAT5
isotig59974	3.19	AT3G57230.1	AGL16	Agamous-like MADS-box protein AGL16
isotig33091	3.18	AT1G14920.1	GAI	DELLA protein GAI
isotig39479	3.15	AT2G21660.1	RBG7	Glycine-rich RNA-binding protein 7
isotig27037	3.07	AT2G35060.1	POT11	Potassium transporter 11
isotig64424	2.7	AT3G58850.1	PAR2	Transcription factor PAR2
isotig56444	2.60	AT3G12810.1	PIE1	Protein PHOTOPERIOD-INDEPENDENT EARLY FLOWERING 1
isotig43989	2.53	AT1G26780.1	MYB117	myb domain protein 117
isotig56396	2.47	AT3G51550.1	FER	Receptor-like protein kinase FERONIA
isotig61074	2.44	AT1G18750.1	AGL65	AGAMOUS-like 65
isotig40387	2.42	AT1G52150.1	ATHB-15	Homeobox-leucine zipper protein ATHB-15
isotig58190	2.42	AT1G32240.1	KAN2	Probable transcription factor KAN2
isotig58719	2.38	AT4G04885.1	PCFS4	Polyadenylation and cleavage factor homolog 4
isotig61477	2.36	AT1G06040.1	BBX24	B-box zinc finger protein 24
isotig82050	2.3	AT2G27230.1	LHW	Transcription factor LHW
isotig38826	2.19	AT4G18770.1	MYB98	Transcription factor MYB98
isotig39222	2.15	AT5G59340.1	WOX2	WUSCHEL-related homeobox 2
isotig34304	*-99*	AT1G52150.2	ATHB-15	Homeobox-leucine zipper protein ATHB-15
isotig39945	*-5.57*	AT3G24650.1	ABI3	B3 domain-containing transcription factor ABI3
isotig37162	*-4.92*	AT3G24140.1	FAMA	Transcription factor FAMA
isotig38812	*-4.34*	AT3G59420.1	ACR4	Serine/threonine-protein kinase-like protein ACR4
isotig35426	*-4.18*	AT2G42560.1		LEA domain-containing protein
isotig63058	*-3.34*	AT2G40220.1	ABI4	Ethylene-responsive transcription factor ABI4
isotig47939	*-3.34*	AT1G52880.1	NAC18	NAC domain-containing protein 18

isotig63714	*-3.24*	AT1G77450.1	anac32	NAC domain containing protein 32
isotig63554	*-3.10*	AT2G40220.1	ABI4	Ethylene-responsive transcription factor ABI4
isotig94895	*-3.1*	AT1G19220.1	ARF19	Auxin response factor 19
isotig65664	*-2.96*	AT1G20930.1	CDKB2-2	Cyclin-dependent kinase B2-2
isotig63792	*-2.75*	AT1G01720.1	NAC2	NAC domain-containing protein 2
isotig41211	*-2.72*	AT1G51190.1	PLT2	AP2-like ethylene-responsive transcription factor PLT2
isotig33112	*-2.60*	AT1G05230.3	HDG2	Homeobox-leucine zipper protein HDG2
isotig61378	*-2.53*	AT3G57670.1	WIP2	Zinc finger protein WIP2
isotig07912	*-2.41*	AT4G30270.1	XTH24	Xyloglucan ndotransglucosylase/hydrolase protein 24
isotig11918	*-2.12*	AT4G31920.1	ARR1	Two-component response regulator ARR10
isotig81524	*-2.09*	AT2G21660.1	RBG7	Glycine-rich RNA-binding protein 7
isotig19043	*-2.07*	AT3G08970.1	ERDJ3A	DnaJ protein ERDJ3A
isotig84240	*-2.06*	AT2G21660.1	RBG7	Glycine-rich RNA-binding protein 7
isotig62912	*-2.04*	AT3G54180.1	CDKB1-1	Cyclin-dependent kinase B1-1
**Pairwise comparison E2/E3DO**				
isotig71762	2.09	AT1G51450.1	TRO	Protein TRAUCO
isotig50769	*-2.14*	AT5G59340.1	WOX2	WUSCHEL-related homeobox 2
isotig25191	-2.14	AT5G59340.1	WOX2	WUSCHEL-related homeobox 2
**Pairwise comparison E3DO/E4**				
isotig101628	99	AT5G62000.1	ARF2	Auxin response factor 2
isotig89845	99	AT4G32551.1	LUG	Transcriptional corepressor LEUNIG
isotig50769	4.46	AT5G59340.1	WOX2	WUSCHEL-related homeobox 2
isotig13246	3.91	AT5G59340.1	WOX2	WUSCHEL-related homeobox 2
isotig25191	3.47	AT5G59340.1	WOX2	WUSCHEL-related homeobox 2
isotig04574	3.36	AT5G09970.1	CYP78A7	Cytochrome P450 78A7
isotig65385	3.21	AT1G52880.1	NAC018	NAC domain-containing protein 18
isotig67346	3.14	AT5G15540.1	EMB2773	PHD finger family protein
isotig61540	2.63	AT3G15510.1	ATNAC2	NAC domain containing protein 2
isotig18917	2.41	AT3G04070.2	anac047	NAC domain containing protein 47
isotig45225	2.35	AT2G30130.1	LBD12	LOB domain-containing protein 12
isotig71987	*-99*	AT1G03120.1	ATRAB28	responsive to abscisic acid 28
isotig71994	*-99*	AT2G01500.1	WOX6	WUSCHEL-related homeobox 6
isotig71508	*-4.82*	AT2G30420.1	ETC2	MYB-like transcription factor ETC2
isotig39159	*-2.89*	AT1G03120.1	ATRAB28	responsive to abscisic acid 28
isotig37135	*-2.87*	AT4G08150.1	KNAT1	Homeobox protein knotted-1-like 1
isotig59371	*-2.54*	AT5G09970.1	CYP78A7	Cytochrome P450 78A7
isotig59013	*-2.40*	AT3G13960.1	GRF5	Growth-regulating factor 5
isotig63158	*-2.40*	AT1G26870.1	ANAC009	NAC domain transcriptional regulator superfamily protein
isotig60977	*-2.38*	AT5G61850.1	LFY	Protein LEAFY
isotig09216	-2.11	AT5G54380.1	THE1	Receptor-like protein kinase THESEUS 1

**Pairwise comparison E3DO/E3SU**				
isotig25191	5.57	AT5G59340.1	WOX2	WUSCHEL-related homeobox 2
isotig50769	4.57	AT5G59340.1	WOX2	WUSCHEL-related homeobox 2
isotig18917	4.09	AT3G04070.2	anac047	NAC domain containing protein 47
isotig57427	2.73	AT5G63090.2	LOB	Protein LATERAL ORGAN BOUNDARIES
isotig14005	2.59	AT1G65910.1	anac028	NAC domain containing protein 28
isotig62449	2.51	AT2G40220.1	ABI4	Ethylene-responsive transcription factor ABI4
isotig13246	2.43	AT5G59340.1	WOX2	WUSCHEL-related homeobox 2
isotig61540	2.41	AT3G15510.1	ATNAC2	NAC domain containing protein 2
isotig65385	2.32	AT1G52880.1	NAC018	NAC domain-containing protein 18
isotig65643	2.17	AT3G04070.2	anac047	NAC domain containing protein 47
isotig71508	*-3.40*	AT2G30420.1	ETC2	MYB-like transcription factor ETC2
isotig59371	-2.54	AT5G09970.1	CYP78A7	Cytochrome P450 78A7
