# Supplementary material for: Transcript profiling for early stages during embryo development in Scots pine
Source: BMC Plant Biol. 2016 Nov 18;16:255. doi: 10.1186/s12870-016-0939-5 (PMC5116219; doi:10.1186/s12870-016-0939-5)
Supplement: Additional file 11: — Figure S8. Clustering of differentially expressed transcripts identified in the pairwise comparisons between different megagametophyte developmental stages. The analysis included DETs with a FC > 2 and RPKM > 10. For each transcript, RPKM values were normalized to its maximum RPKM value during development. Normalized values were subjected to k-means clustering method and classified into different clusters, based on their expression levels across the four developmental stages. The Y-axis of the cluster chart represents relative expression (from 0 to 1). The percentage of transcripts with GO terms assigned to different Biological Processes (GO at level 2) for each type is shown. Figure S9. Differentially expressed transcripts between consecutive stages in megagametophytes during early seed development. The analysis included DETs, with FC > 2 and RPKM > 10, identified in any of the three pairwise comparisons. (A) Histogram showing the number of up- and down-regulated DETs between different developmental stages. (B) Venn diagram showing the common and specific number of DETs detected in the consecutive pairwise analysis. (C) Summary table showing the number of DETs (excluding those with no assigned matches against TAIR) involved in different processes in each development transition. (PDF 1.23 kb) [file 12870_2016_939_MOESM11_ESM.pdf]

Figure S8. Clustering of differentially expressed transcripts identified in the pairwise comparisons between different megagametophyte developmental stages. The analysis included DETs with a FC>2 and RPKM>10. For each transcript, RPKM values were normalized to its maximum RPKM value during development. Normalized values were subjected to k-means clustering method and classified into different clusters, based on their expression levels across the four developmental stages. The Y-axis of the cluster chart represents relative expression (from 0 to 1). The percentage of transcripts with GO terms assigned to different Biological Processes (GO at level 2) for each type is shown.

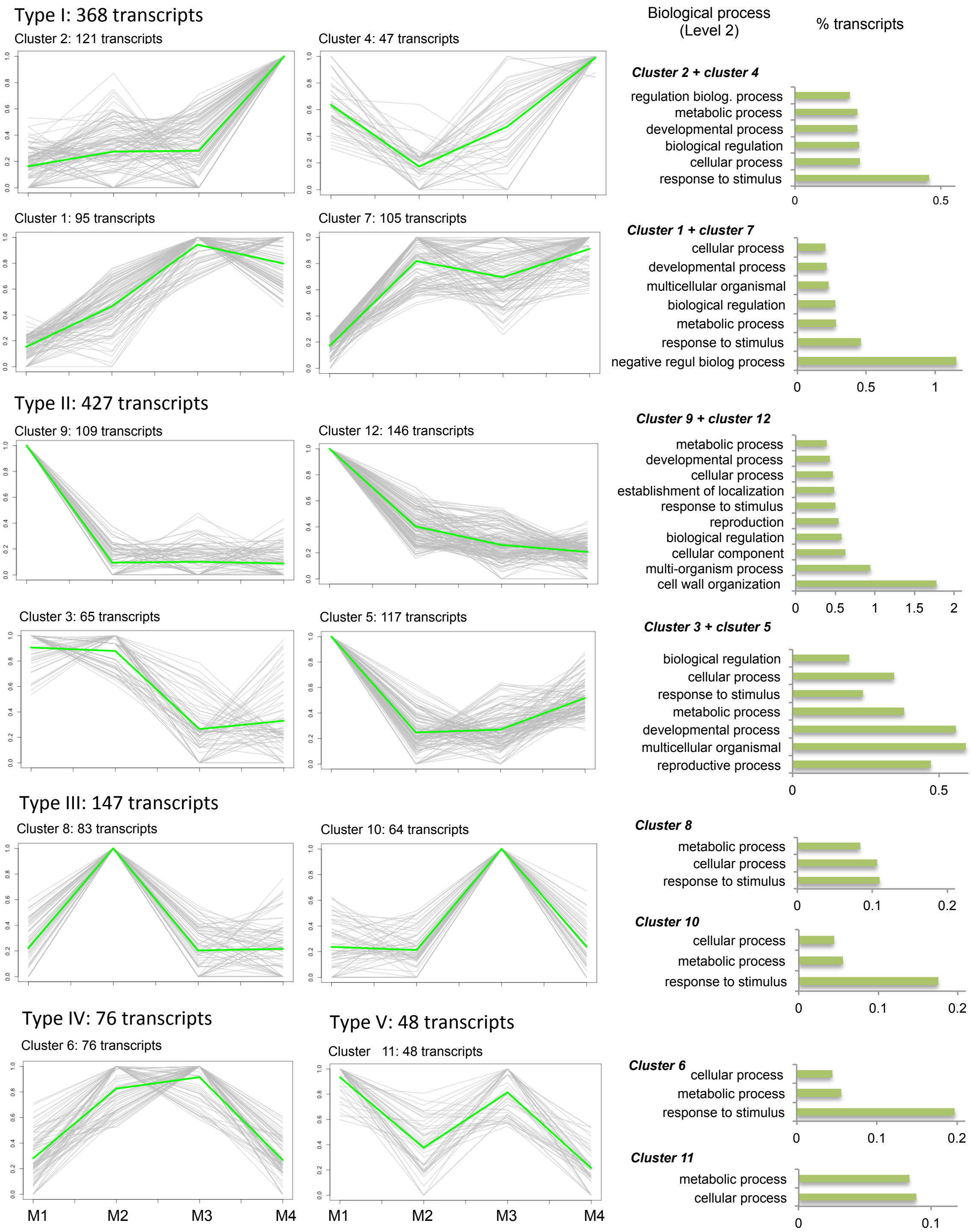

Figure S9. Differentially expressed transcripts between consecutive stages in megagametophytes during early seed development. The analysis included DETs, with FC>2 and RPKM>10, identified in any of the three pairwise comparisons. (A) Histogram showing the number of up- and down-regulated DETs between different developmental stages. (B) Venn diagram showing the common and specific number of DETs detected in the consecutive pairwise analysis. (C) Summary table showing the number of DETs (excluding those with no assigned matches against TAIR) involved in different processes in each development transition.

A

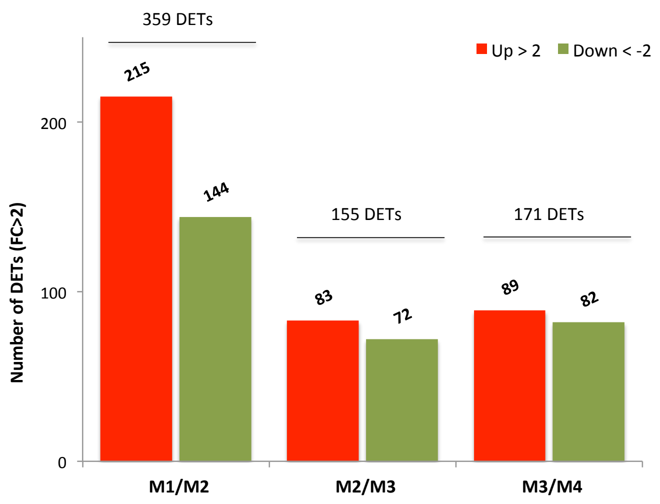

B

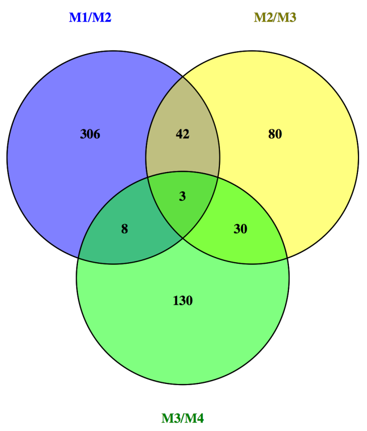

C

	M1/M2		M2/M3		M3/M4	
Transcript regulation	Up	Down	Up	Down	Up	Down
Number of DETs	215	144	83	72	89	82
Number of differentially expressed TFs	13	6	3	2	2	3
Transcripts with assigned GO terms related to developmental process	10	3	3	3	1	2
Transcripts with assigned GO terms related to response to stress and stimulus	13	8	7	9	2	12
